# Alterations in gut microbiota composition in neurodevelopmental disorders: a systematic review and meta-analysis

**DOI:** 10.3389/fmicb.2025.1650212

**Published:** 2025-12-09

**Authors:** Hua Yang, Anqi Wang, Jie Yang, Rong Luo, Yue Yang

**Affiliations:** 1 Department of Pediatrics, West China Second University Hospital, Sichuan University, Chengdu, Sichuan, China; 2 Key Laboratory of Birth Defects and Related Diseases of Women and Children (Sichuan University), Ministry of Education, Chengdu, Sichuan, China; 3 Sichuan Provincial Center for Mental Health, Sichuan Academy of Medical Sciences and Sichuan Provincial People’s Hospital, Chengdu, Sichuan, China; 4 Key Laboratory of Psychosomatic Medicine, Chinese Academy of Medical Sciences, Chengdu, Sichuan, China

**Keywords:** gut microbiota, dysbiosis, neurodevelopmental disorders, humans, systematic review and meta-analysis

## Abstract

**Background:**

Neurodevelopmental disorders (NDDs) have been linked to changes in the gut microbiome, but the exact nature of these alterations is not fully understood. This research seeks to explore the variations in both the diversity and composition of the gut microbiota in individuals diagnosed with NDDs.

**Methods:**

We conducted a systematic literature search up to April 2025. Meta-analyses using STATA 16.0 evaluated alpha diversity, beta diversity, and relative abundance between individuals with NDDs and healthy controls.

**Results:**

No significant alpha diversity differences were found between NDD and control groups. Beta diversity analysis revealed distinct microbial communities across autism spectrum disorder (ASD), attention-deficit/hyperactivity disorder (ADHD), and tic disorder (TD) subgroups. At the family level, NDDs showed increased Peptostreptococcaceae (SMD = 0.47; 95% CI: 0.05 to 0.90). Genus-level analysis demonstrated reduced *Escherichia/Shigella* (SMD = −0.39; 95% CI: −0.59 to −0.19) and *Roseburia* (SMD = −0.39; 95% CI: −0.78 to 0), alongside elevated *Eubacterium* (SMD = 0.33; 95% CI: 0.20–0.47) in NDDs.

**Conclusion:**

This study highlights the complex changes in gut microbiota in NDDs, particularly significant differences at the beta diversity, family, and genus levels. However, the results are constrained by research heterogeneity and small sample sizes. To better elucidate these associations, larger, more standardized studies are required.

**Systematic review registration:**

https://www.crd.york.ac.uk/prospero/, CRD42024585913.

## 1 Introduction

Neurodevelopmental disorders (NDDs) comprise a group of conditions characterized by impaired brain development and function, leading to cognitive, emotional, and behavioral disturbances. Affecting approximately 10% of children worldwide (Dash et al., 2022; Morris-Rosendahl and Crocq, 2020; Thapar et al., 2017), NDDs represent a heterogeneous collection of conditions including autism spectrum disorder (ASD), attention-deficit/hyperactivity disorder (ADHD), tic disorders (TD), intellectual disability, and developmental coordination disorder (Lukens and Eyo, 2022; American Psychiatric Association, 2013). Their etiology involves complex interactions between genetic, environmental, and epigenetic factors (Lord et al., 2018; Dall’Aglio et al., 2018; Thapar et al., 2013). In recent years, the gut-brain axis has emerged as a key pathophysiological mechanism and research focus in NDDs.

The gut microbiota constitutes a complex ecosystem of microorganisms residing in the human gastrointestinal tract (Clarke et al., 2014). This community plays crucial roles in nutrient metabolism, bioactive compound production, and the regulation of host physiology, including immune function, neural activity, and intestinal barrier integrity (Cryan et al., 2019; De Theije et al., 2014; Borre et al., 2014). The gut-brain axis provides a bidirectional communication network between gut microbes and the central nervous system, enabling microbial influence on brain function and behavior (Chernikova et al., 2021; Goncalves et al., 2024). This connection is particularly relevant in NDDs, where patients frequently experience both gastrointestinal symptoms and neuropsychiatric manifestations.

ASD, among the most prevalent NDDs, demonstrates substantial gut microbiota alterations compared to neurotypical individuals. Multiple investigations have revealed distinct differences in microbial community structure and composition. Kang et al. (2013) reported reduced abundance of *Prevotella* and *Coprococcus* in children with ASD, particularly those presenting gastrointestinal symptoms. Bezawada et al. (2020) documented elevated levels of *Clostridium* and *Desulfovibrio*, which may promote neuroinflammation through toxin production. Some studies suggest that interventions such as prebiotics, probiotics, or fecal microbiota transplantation, which restore gut microbial balance, may lead to improvements in neurofunctional and behavioral symptoms (Martinez-Gonzalez and Andreo-Martinez, 2020; Tan et al., 2021; Song et al., 2022).

In ADHD, considerable gut microbiota differences relative to healthy controls have been consistently observed. These include reduced microbial diversity and altered taxonomic profiles, notably imbalances in the Firmicutes/Bacteroidetes ratio (Prehn-Kristensen et al., 2018). Increased *Lactobacillus* abundance has been associated with ADHD symptomatology, potentially through modulation of neurotransmitter systems (e.g., serotonin, dopamine) and gut-brain axis-mediated neuroinflammation (Bull-Larsen and Mohajeri, 2019; Aarts et al., 2017). Furthermore, Aresti-Sanz et al. (2021) demonstrated that ADHD medications such as methylphenidate can modify gut microbiota composition, adding complexity to microbiome study interpretations. Dietary interventions including vitamin D3 supplementation and probiotic administration have yielded modest symptomatic benefits, reinforcing the gut-brain connection in ADHD (Abhishek et al., 2024).

Although research is still limited, emerging evidence suggests that the gut microbiota may modulate the neurobiological mechanisms underlying TD. TD shows differences in gut microbiota composition compared to healthy controls (Xi et al., 2021; Wang et al., 2022). Dysbiosis may exacerbate neuroinflammation or affect dopamine signaling, potentially playing a role in TD pathogenesis (Altaib et al., 2021; Kanaan et al., 2017; Nikolaus et al., 2022; De Jong et al., 2016). Xi et al. (2021) identified increased *Escherichia/Shigella* in TD patients, possibly linked to dopamine dysregulation. Geng et al. (2023) reported elevated pro-inflammatory bacteria (e.g., *Bacteroides*), supporting the neuroinflammation hypothesis in TD. Many Tourette syndrome (TS) patients also experience gastrointestinal symptoms, further supporting the link between gut microbiota and TD.

Although previous studies have documented alterations in gut microbiota within individual NDDs, a quantitative meta-analysis that concurrently integrates cohorts from ASD, ADHD, and TD is still lacking. The present study was therefore designed to address this gap by systematically consolidating and statistically analyzing gut microbiome variations across these three major NDDs. Our objectives are to identify robust, clinically relevant microbial biomarkers and to provide informed recommendations for future research in this rapidly evolving field.

This study is registered with PROSPERO, https://www.crd.york.ac.uk/prospero/, under the ID number CRD42024585913.

## 2 Methods

### 2.1 Search strategy

This systematic review and meta-analysis was conducted in accordance with PRISMA guidelines (Page et al., 2021). On April 8, 2025, we performed a comprehensive literature search across multiple databases including PubMed, Embase, Cochrane Library, Web of Science, Scopus, and PsycINFO. The complete search strategy is provided in Supplementary Table S1.

### 2.2 Inclusion and exclusion criteria

Eligibility for study inclusion was determined according to the following predefined parameters: (1) Case–control study design; (2) Study populations comprising patients with clinically diagnosed NDDs (ASD, ADHD, or TD); (3) Comparative analyses of gut microbiota composition between NDD patients and healthy controls; (4) Reporting of quantitative gut microbial diversity metrics (alpha- or beta-diversity indices) and/or relative abundance data; (5) Microbiota profiling using fecal samples. Studies were excluded according to the following criteria: (1) Animal model or *in vitro* studies; (2) Investigations analyzing non-fecal samples (e.g., blood, urine) or reporting only microbial metabolites without microbiota composition data; (3) Literature reviews, meta-analyses, case reports, conference abstracts, or editorials; (4) Non-English publications.

### 2.3 Data extraction

Two investigators (HY and AW) independently extracted data using a standardized form. Extracted information included publication details, participant demographics, clinical characteristics, and methodological parameters. We also documented whether studies accounted for dietary factors or the use of probiotics and antibiotics. Primary outcomes encompassed gut microbiota characteristics, including community-level alpha/beta diversity and taxonomic composition (from phylum to genus levels). Any discrepancies between reviewers were resolved through discussion with a third investigator (JY). Corresponding authors were contacted for additional data when necessary.

Study quality was assessed using the Newcastle-Ottawa Scale (NOS), which evaluates three domains (selection, comparability, exposure) across eight items. The maximum achievable scores were 4, 2, and 3 points per domain, respectively. Studies scoring ≥7 points were considered high quality.

### 2.4 Statistical analysis

Statistical analyses were conducted in STATA 16.0. Microbial community characteristics were evaluated through alpha-diversity, beta-diversity, and hierarchical taxonomic profiling (phylum to genus). Data transformation from medians (IQR) to means (SD) was performed using established computational methods.^1^

Numerical data were extracted from graphical representations using GetData Graph Digitizer software (version 2.26) and Adobe Acrobat’s measurement tool when required. Continuous variables were expressed as standardized mean differences (SMD) with 95% confidence intervals (CI) to evaluate effect sizes and between-study variability.

The I^2^ statistic was used to assess heterogeneity of effect sizes, with values categorized as low (25%), moderate (50%), or high (75%) heterogeneity. Sensitivity analyses were conducted to evaluate result robustness and identify potential sources of heterogeneity. Publication bias was assessed using Egger’s regression test and funnel plot inspection. Statistical significance was defined as *p* < 0.05 for all analyses.

## 3 Results

### 3.1 Search results

**Figure 1:**
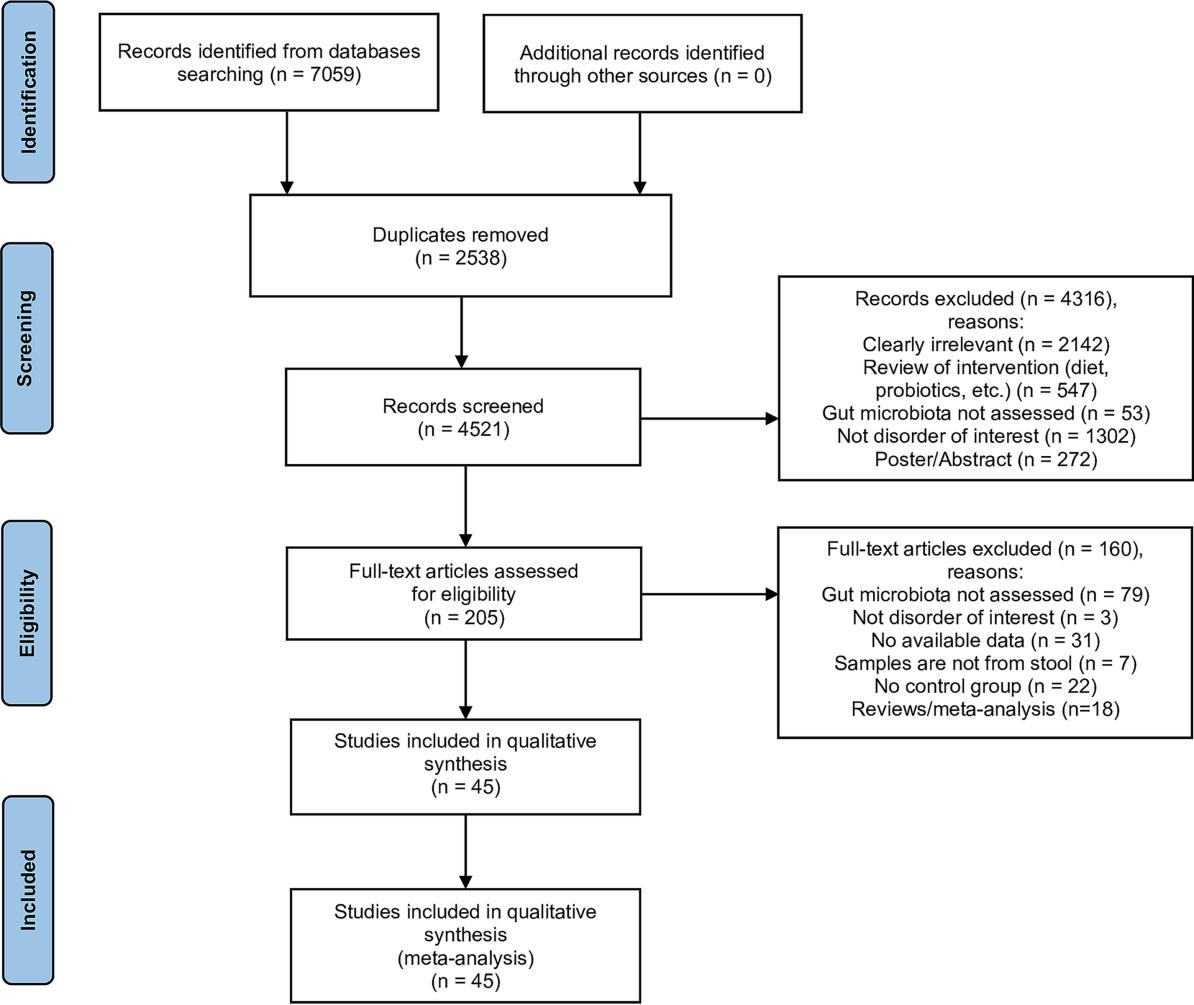
Flow diagram for selection of studies (PRISMA flow diagram).

Our systematic search identified 7,059 records from multiple databases: PubMed (*n* = 2,628), Scopus (*n* = 1,516), Web of Science (*n* = 1,436), Embase (*n* = 750), PsycINFO (*n* = 498), and Cochrane Library (*n* = 231). Following removal of 2,538 duplicates, we screened 4,521 records based on title and abstract. Of these, 205 full-text articles were assessed for eligibility. We excluded 79 studies that did not assess gut microbiota, 22 lacking control groups, 31 with insufficient data, 18 review articles or meta-analyses, 7 utilizing non-fecal samples, and 3 focusing on non-target disorders (Figure 1). The final analysis included 45 case–control studies published between 2011 and 2025. Figure 1 illustrates the study selection process, and the PRISMA checklist is provided in Supplementary Table S2.

### 3.2 Characteristics of included studies

The 45 included studies comprised 2,767 NDD patients and 1,611 age-matched neurotypical controls. A pooled analysis of all participants revealed the following ranges across the individual studies: age, 2–33 years; male proportion, 37.5–100%; and BMI, 14.7–24.7 kg/m^2^ (Table 1). The majority of the research was carried out in China, representing 26 studies (57.8%) (Wang et al., 2022; Zhang et al., 2018; Sun et al., 2019; Ma et al., 2019; Niu et al., 2019; Zou et al., 2020; Ding et al., 2020; Chen et al., 2020; Cao et al., 2021; Wan et al., 2022; Ye et al., 2021; Huang et al., 2021; Chen et al., 2021; Ding et al., 2021; Chen et al., 2022; Deng et al., 2022; He et al., 2023; Wang et al., 2023; Zhao et al., 2023; Mendive Dubourdieu and Guerendiain, 2023; Pang et al., 2023; Yitik Tonkaz et al., 2023; Xu and Zhang, 2023; Li et al., 2023; Jiang et al., 2018; Wang et al., 2020; Wan et al., 2020; Bao et al., 2023). Italy (Strati et al., 2017; Coretti et al., 2018; Chiappori et al., 2022) and Thailand (Bhusri et al., 2025; Panpetch et al., 2024; Boonchooduang et al., 2025) each contributed 3 studies (6.7%). Spain (Plaza-Diaz et al., 2019; Richarte et al., 2021) and the Netherlands (Aarts et al., 2017; Szopinska-Tokov et al., 2020) provided 2 studies each (4.4%), with single studies from Australia (Wang et al., 2011), America (Kang et al., 2013), India (Pulikkan et al., 2018), Russia (Kovtun et al., 2020), Denmark (Bundgaard-Nielsen et al., 2023), Uruguay (Mendive Dubourdieu and Guerendiain, 2023), Turkey (Yitik Tonkaz et al., 2023), Germany (Prehn-Kristensen et al., 2018), and Israel (Steckler et al., 2024) (2.2% each).

**Table 1:** Characteristics of the studies included in the meta-analysis.

Study	Disorder	Country	Definition of disorder	Sample Size, n	Mean Age, years	Male, %	Mean BMI	Dietary Assessment	Probiotics Usage	Antibiotics Usage	Sequencing	Diversity Assessments
Wang et al. (2011)	ASD	Australia	DSM-IV	P: 23 HC: 9	P: 10.3 HC: 9.5	P: 91.3 HC: 44.4	–	–	–	–	qPCR	α: not measured *β*: not measured
Kang et al. (2013)	ASD	America	ADI-Revised, ADOS	P: 20 HC: 20	P: 6.7 HC: 8.3	P: 90.0 HC: 85.0	–	YES	NO	–	Pyrosequencing	α: Chao1, Shannon, PD β: UniFrac (weighted)
Strati et al. (2017)	ASD	Italy	DSM-5	P: 40 HC: 40	P: 11.1 HC: 9.2	P: 77.5 HC: 70.0	–	YES	NO	NO	16S rRNA V3-V5	α: not measured β: UniFrac (weighted & unweighted), Bray-Curtis
Pulikkan et al. (2018)	ASD	India	DSM-5	P: 30 HC: 24	P: 9.5 HC: 9.5	P: 93.3 HC: 62.5	P: 14.8 HC: 15.8	–	–	NO	16S rRNA V3	α: Observed sp., Shannon, PD *β*: UniFrac (unweighted)
Zhang et al. (2018)	ASD	China	DSM-5	P: 35 HC: 6	P: 4.9 HC: 4.6	P: 82.9 HC: 83.3	–	–	NO	NO	16S rRNA	α: Shannon β: UniFrac (weighted), Bray-Curtis
Coretti et al. (2018)	ASD	Italy	DSM-5	P: 11 HC: 14	P: 2.9 HC: 2.9	P: 81.8 HC: 57.1	–	YES	–	NO	16S rRNA V3-V4	α: Observed sp., Shannon, Goods coverage β: UniFrac (weighted & unweighted)
Sun et al. (2019)	ASD	China	ICD-11	P: 9 HC: 6	P: 3.0–12.0 HC: 3.0–12.0	P: 88.9 HC: 66.7	–	–	–	–	16S rRNA V3-V4	α: Shannon, Simpson β: not measured
Plaza-Diaz et al. (2019)	ASD	Spain	DSM-5	P: 48 HC: 57	P: 3.7 HC: 4.3	-	P: 15.9 HC: 16.3	YES	–	–	16S rRNA V3-V4	α: not measured β: not measured
Ma et al. (2019)	ASD	China	DSM-5	P: 45 HC: 45	P: 7.0 HC: 7.3	P: 86.7 HC: 86.7	–	YES	NO	NO	16S rRNA V3-V4	α: Chao1, Shannon, ACE, PD β: UniFrac (weighted & unweighted), Bray-Curtis
Niu et al. (2019)	ASD	China	DSM-5	P: 114 HC: 40	P: 4.5 HC: 4.2	P: 83.3 HC: 50.0	–	–	NO	NO	16S rRNA V3-V4	α: Shannon, Simpson β: not measured
Zou et al. (2020)	ASD	China	DSM-IV	P: 48 HC: 48	P: 5.0 HC: 4.0	P: 79.17 HC: 50.0	P: 17.4 HC: 16.3	–	NO	NO	16S rRNA V3-V4	α: Chao1, Shannon, ACE, Shannon evenness, Goods coverage β: UniFrac (weighted)
Ding et al. (2020)	ASD	China	DSM-5	P: 77 HC: 50	P: 3.2 HC: 3.6	P: 76.6 HC: 78.0	–	YES	NO	NO	16S rRNA V4	α: Observed sp., Chao1, Shannon β: UniFrac (weighted & unweighted), Bray-Curtis
Kovtun et al. (2020)	ASD	Russia	DSM-5	P: 30 HC: 20	P: 3.0–5.0 HC: 3.0–5.0	P: 86.7 HC: 70.0	–	–	NO	NO	Shotgun Metagenomics	α: Chao1, Shannon, ACE, Simpson β: not measured
Chen et al. (2020)	ASD	China	CARS, ADI-R, ADOS	P: 76 HC: 47	P: 4.0 HC: 4.3	P: 80.3 HC: 87.2	P: 15.8 HC: 16.4	YES	NO	NO	16S rRNA V3-V4	α: Observed sp., β: Bray-Curtis
Cao et al. (2021)	ASD	China	DSM-5	P: 45 HC: 41	P: 6.8 HC: 5.2	P: 80.0 HC: 82.9	P: 16.6 HC:15.2	–	NO	NO	16S rRNA V4	α: Shannon β: Bray-Curtis
Wan et al. (2022)	ASD	China	DSM-5	P: 64 HC: 64	P: 4.9 HC: 4.7	P: 82.8 HC: 83.1	–	YES	NO	NO	16S rRNA	α: Chao1, Shannon β: UniFrac (weighted & unweighted), Bray-Curtis
Ye et al. (2021)	ASD	China	DSM-5	P: 71 HC: 18	P: 4.3 HC: 4.6	P: 100.0 HC: 100.0	–	–	NO	NO	16S rRNA V1-V2, Metagenomic sequencing	α: Observed sp., Chao1, Shannon, Simpson β: UniFrac (weighted)
Huang et al. (2021)	ASD	China	DSM-5	P: 39 HC: 44	P: 4.7 HC: 5.1	P: 82.1 HC: 70.5	–	–	NO	NO	16S rRNA V4-V5	α: Shannon, Inv. Simpson β: UniFrac (weighted & unweighted), Bray-Curtis
Chen et al. (2021)	ASD	China	DSM-5	P: 138 HC: 60	P: 6.1 HC: 6.7	P: 84.8 HC: 45.0	P: 20.9 HC: 17.7	–	–	–	16S rRNA V3-V4	α: Observed sp., Chao1, Shannon, Inv. Simpson β: UniFrac (weighted & unweighted), Bray-Curtis
Ding et al. (2021)	ASD	China	DSM-5	P: 25 HC: 20	P: 5.7 HC: 5.4	P: 84.0 HC: 60.0	P: 21.9 HC: 24.3	–	NO	NO	16S rRNA	α: Chao1, Shannon, ACE β: UniFrac (unweighted)
Chen et al. (2022)	ASD	China	DSM-5	P: 82 HC: 31	P: 17.2 HC: 13.0	P: 100.0 HC: 100.0	P: 17.7 HC: 17.1	–	–	–	16S rRNA V3-V4	α: Shannon, Simpson, PD, Goods coverage β: UniFrac (weighted & unweighted)
Deng et al. (2022)	ASD	China	DSM-5	P: 45 HC: 45	P: 6.0 HC: 6.1	P: 86.7 HC: 46.7	P: 16.2 HC: 15.6	YES	NO	NO	16S rRNA V3-V5	α: Observed sp., Chao1, Shannon, Simpson, ACE, PD β: UniFrac (weighted & unweighted), Bray-Curtis
Chiappori et al. (2022)	ASD	Italy	DSM-5	P: 6 HC:6	P: 6.0–17.0 HC: 10.0–20.0	P: 83.3 HC: 50.0	–	–	–	–	16S rRNA V3-V4	α: Shannon, Simpson β: UniFrac (weighted & unweighted), Bray-Curtis
He et al. (2023)	ASD	China	DSM-5	P: 40 HC: 40	P: 5.3 HC: 5.8	P: 75.0 HC: 67.5	P: 14.7 HC: 15.0	YES	NO	NO	16S rRNA V3-V4	α: Observed sp., Chao1, Shannon, Simpson, ACE, J Index β: not measured
Bundgaard-Nielsen et al. (2023)	ASD	Denmark	ICD-10	P: 12 HC: 17	P: 12.0 HC: 10.0	P: 75.0 HC: 52.9	–	YES	–	NO	16S rRNA V4	α: ASV Richness, Shannon, PD β: UniFrac (weighted & unweighted), Bray-Curtis
Wang et al. (2023)	ASD	China	DSM-5	P: 42 HC: 41	P: 5.8 HC: 6.8	P: 66.7 HC: 61.0	–	–	NO	NO	16S rRNA	α: Observed sp., Chao1, Shannon, Simpson, ACE, J Index β: not measured
Zhao et al. (2023)	ASD	China	DSM-5	P: 36 HC: 40	P: 3.9 HC: 4.3	P: 75.0 HC: 77.5	–	YES	NO	NO	16S rRNA V3-V4	α: Observed sp., Shannon β: UniFrac (weighted & unweighted), Bray-Curtis
Mendive Dubourdieu and Guerendiain (2023)	ASD	Uruguay	DSM-5	P: 30 HC: 28	P: 3.0–12.0 HC: 3.0–12.0	-	–	YES	NO	NO	16S rRNA V1-V9	α: not measured β: not measured
Pang et al. (2023)	ASD	China	ICD-10	P: 19 HC: 19	P: 21.0 HC: 29.0	P: 73.7 HC: 43.3	P: 21.9 HC: 24.3	-	NO	NO	16S rRNA V3–V4	α: Chao1, Shannon, Simpson β: Bray-Curtis
Yitik Tonkaz et al. (2023)	ASD	Turkey	DSM-5	P: 30 HC: 30	P: 7.3 HC: 8.5	P: 86.7 HC:43.3	P: 17.7 HC: 17.1	YES	NO	NO	qPCR	α: not measured β: not measured
Xu and Zhang (2023)	ASD	China	DSM-5	P: 10 HC: 10	P: 4.8 HC: 4.2	P: 80.0 HC: 80.0	–	YES	NO	NO	16S rRNA V4	α: Observed sp., Chao1, Shannon, Simpson, ACE β: not measured
Li et al. (2023)	ASD	China	DSM-5	P: 957 HC: 161	P: 4.6 HC: 4.8	P: 70.7 HC: 75.8	–	–	NO	NO	16S rRNA V3-V4	α: Chao1, Shannon, Simpson β: UniFrac (weighted & unweighted), Bray-Curtis
Bhusri et al. (2025)	ASD	Thailand	ADOS	P: 62 HC: 33	P: 19.0 HC: 12.0	P: 56.5 HC: 90.9	–	–	NO	NO	16S rRNA V4	α: Shannon, Simpson, ASV Richness β: Bray-Curtis
Aarts et al. (2017)	ADHD	Netherlands	DSM-IV	P: 19 HC: 77	P: 19.5 HC: 27.1	P: 68.4 HC: 53.2	P: 23.8 HC: 23.0	–	–	NO	16S rRNA	α: Observed sp., Chao1, Shannon, PD β: not measured
Jiang et al. (2018)	ADHD	China	DSM-IV	P: 51 HC: 32	P: 8.47 HC: 8.5	P: 74.5 HC:37.5	P: 16.4 HC: 16.1	–	–	–	16S rRNA V3-V4	α: Chao1, Shannon, Simpson β: UniFrac (weighted & unweighted), Bray-Curtis
Prehn-Kristensen et al. (2018)	ADHD	Germany	DSM-IV	P: 14 HC: 17	P: 11.9 HC: 13.1	P: 100.0 HC: 100.0	P: 19.0 HC: 18.0	–	–	–	16S rRNA V1-V2	α: Observed sp., Chao1, Shannon β: not measured
Szopinska-Tokov et al. (2020)	ADHD	Netherlands	DSM-IV	P: 41 HC: 48	P: 20.2 HC: 20.5	P: 63.0 HC: 40.0	P: 23.0 HC: 22.0	–	–	–	16S rRNA V1-V2	α: Observed sp., Chao1 β: UniFrac (weighted)
Wang et al. (2020)	ADHD	China	DSM-IV	P: 30 HC: 30	P: 8.4 HC: 9.3	P: 76.7 HC: 60.0	–	YES	NO	NO	16S rRNA V3-V4	α: Chao1, Shannon, Simpson, Goods coverage β: UniFrac (weighted & unweighted)
Wan et al. (2020)	ADHD	China	DSM-5	P: 17 HC: 17	P: 8.0 HC: 8.0	P: 82.3 HC: 76.5	P: 16.1 HC: 15.9	–	–	–	Shotgun Metagenomics	α: Chao1, Shannon, Simpson β: not measured
Richarte et al. (2021)	ADHD	Spain	DSM-5	P: 100 HC: 100	P: 33.0 HC: 30.0	P: 51.0 HC: 47.0	P: 24.7 HC: 22.1	–	NO	NO	16S rRNA V3-V4	α: Observed sp., Shannon, Simpson β: UniFrac (weighted & unweighted), Bray-Curtis
Steckler et al. (2024)	ADHD	Israel	DSM-5	P: 42 HC: 32	P: 11.0 HC: 10.0	P: 85.7 HC: 45.2	–	–	–	NO	16S rRNA V3-V4	α: Observed sp., Shannon, PD β: UniFrac (weighted & unweighted)
Panpetch et al. (2024)	ADHD	Thailand	DSM-5	P: 24 HC: 24	P: 7.0 HC: 7.0	P: 75.0 HC: 75.0	P: 15.0 HC: 15.7	YES	NO	NO	16S rRNA	α: Observed sp., Chao1, Shannon β: UniFrac (weighted & unweighted), Bray-Curtis
Boonchooduang et al. (2025)	ADHD	Thailand	DSM-5	P: 10 HC: 10	P: 6.0–12.0 HC: 6.0–12.0	–	–	–	NO	NO	16S rRNA	α: Observed sp., Shannon, PD, Pielou’s Evenness β: UniFrac (weighted & unweighted), Jaccard
Wang et al. (2022)	TD	China	DSM-5	P: 28 HC: 21	P: 8.2 HC: 7.9	P: 60.7 HC: 61.9	P: 19.3 HC: 18.8	–	NO	NO	16S rRNA V3-V4	α: Chao1, Shannon, Simpson, ACE β: not measured
Bao et al. (2023)	TD	China	DSM-5	P: 32 HC: 29	P: 7.0 HC: 6.4	P: 81.3 HC: 82.8	P: 16.0 HC: 16.2	–	–	NO	16S rRNA	α: Chao1, Shannon, Simpson, β: Bray-Curtis

*ASD, autism spectrum disorder; ADHD, attention deficit hyperactivity disorder; TD, tic disorder; BMI body mass index; P, patient; HC healthy control; DSM, Diagnostic and Statistical Manual of Mental Disorders; ADI-Revised, Autism Diagnostics Interview – Revised; ADOS, Autism Diagnostics Observation Schedule; ICD, International Classification of Diseases; qPCR, quantitative polymerase chain reaction; PD, phylogenetic diversity; Observed* sp.*, observed species; ACE, abundance-based coverage estimator; Inv. Simpson, inverse Simpson*.

Microbiota analyses primarily focused on phylum, family, and genus levels, encompassing diverse bacterial taxa. Bacteroidetes and Firmicutes represented the most abundant phyla in children’s gut microbiota, followed by Actinobacteria. Substantial methodological variations in stool processing and composition analysis were observed across studies (detailed in Table 1; Supplementary Table S3). Dietary factors were evaluated in 17 studies (37.8%), while probiotic use was not reported in 16 studies (35.6%), and antibiotic use was not mentioned in 11 studies (24.4%).

Comprehensive meta-analysis results for bacterial classifications across taxonomic levels are presented in Supplementary Table S4. Forest plots for phylum-level analyses are displayed in the main figures, while non-significant findings for family and genus levels are available in Supplementary Figures S1, S2.

### 3.3 Study quality assessment

Quality assessment using the Newcastle-Ottawa Scale (NOS) classified 44 studies as high quality and 1 study as moderate quality (Supplementary Table S5). Egger’s test results for publication bias are summarized in Supplementary Table S6. Sensitivity analysis demonstrated that pooled effect estimates for all key outcomes remained consistent and were not substantially influenced by any individual study (Supplementary Figure S3).

### 3.4 Alpha diversity

We evaluated 10 different indices measuring richness (Chao1, observed species, abundance coverage estimator, Goods coverage), evenness (Shannon evenness, J Index), combined richness/evenness (Shannon, Simpson, inverse Simpson), and biodiversity (phylogenetic diversity). The most frequently reported indices were Shannon, Chao1, Simpson, abundance coverage estimator (ACE), and observed species.

**Figure 2:**
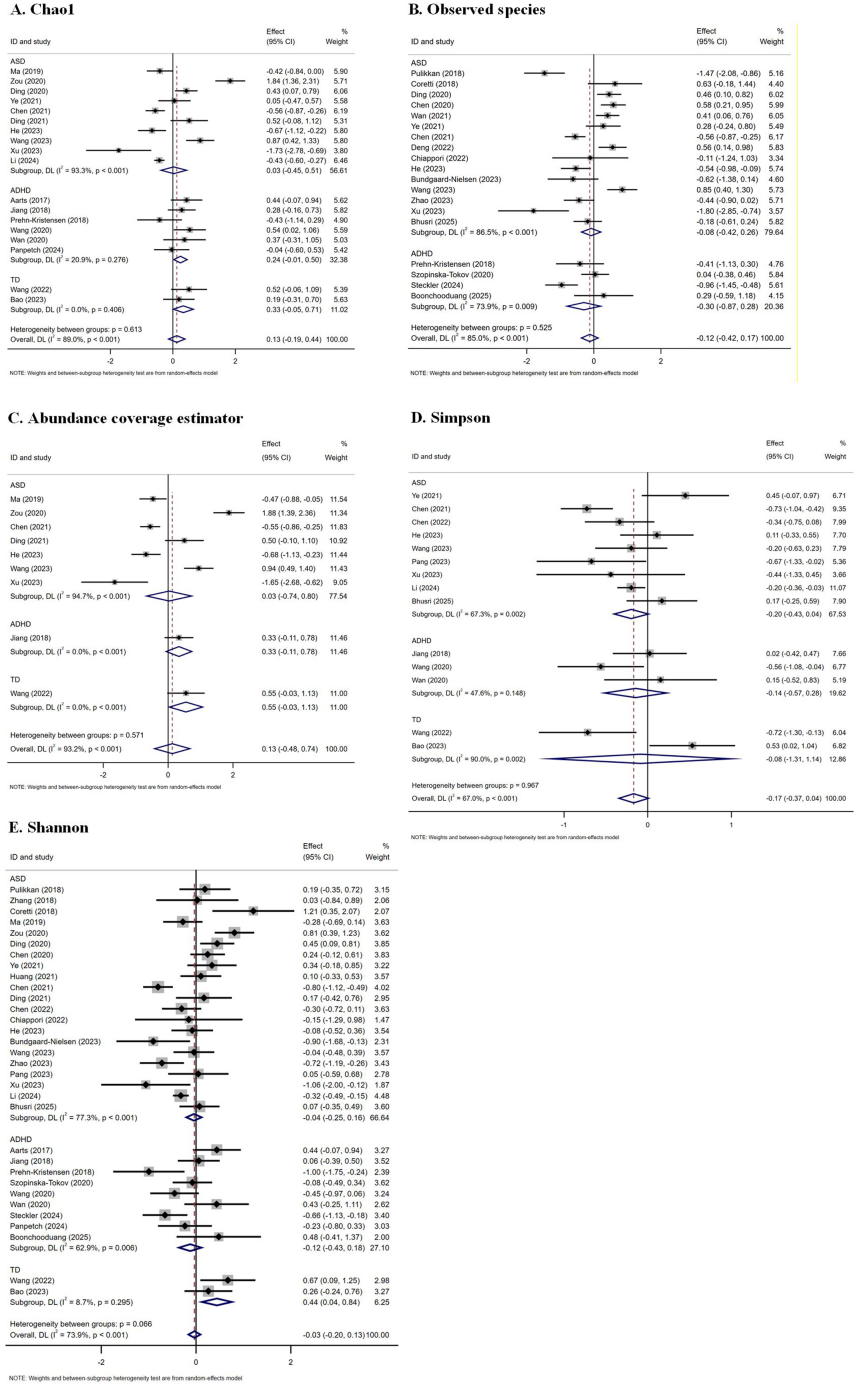
Forest plots of alpha diversity in the gut microbiota of patients with neurodevelopmental disorders compared with healthy controls. **(A)** Chao1; **(B)** Observed species; **(C)** Abundance coverage estimator; **(D)** Simpson; **(E)** Shannon. ASD, autism spectrum disorder; ADHD, attention-deficit/hyperactivity disorder; TD, tic disorder.

A quantitative meta-analysis was conducted on the alpha diversity indices for NDDs and control groups, as shown in Figure 2. Eighteen studies reported the Chao1 index (Prehn-Kristensen et al., 2018; Aarts et al., 2017; Wang et al., 2022; Ma et al., 2019; Zou et al., 2020; Ding et al., 2020; Ye et al., 2021; Chen et al., 2021; Ding et al., 2021; He et al., 2023; Wang et al., 2023; Xu and Zhang, 2023; Li et al., 2023; Jiang et al., 2018; Wang et al., 2020; Wan et al., 2020; Panpetch et al., 2024; Bao et al., 2023), 32 studies reported the Shannon index (Prehn-Kristensen et al., 2018; Aarts et al., 2017; Wang et al., 2022; Pulikkan et al., 2018; Zhang et al., 2018; Coretti et al., 2018; Ma et al., 2019; Zou et al., 2020; Ding et al., 2020; Chen et al., 2020; Ye et al., 2021; Huang et al., 2021; Chen et al., 2021; Ding et al., 2021; Chen et al., 2022; Chiappori et al., 2022; He et al., 2023; Bundgaard-Nielsen et al., 2023; Wang et al., 2023; Zhao et al., 2023; Pang et al., 2023; Xu and Zhang, 2023; Li et al., 2023; Bhusri et al., 2025; Jiang et al., 2018; Szopinska-Tokov et al., 2020; Wang et al., 2020; Wan et al., 2020; Steckler et al., 2024; Panpetch et al., 2024; Boonchooduang et al., 2025; Bao et al., 2023), 14 studies reported the Simpson index (Wang et al., 2022; Ye et al., 2021; Chen et al., 2021; Chen et al., 2022; He et al., 2023; Wang et al., 2023; Pang et al., 2023; Xu and Zhang, 2023; Li et al., 2023; Bhusri et al., 2025; Jiang et al., 2018; Wang et al., 2020; Wan et al., 2020; Bao et al., 2023), 9 studies reported the ACE (Wang et al., 2022; Ma et al., 2019; Zou et al., 2020; Chen et al., 2021; Ding et al., 2021; He et al., 2023; Wang et al., 2023; Xu and Zhang, 2023; Li et al., 2023; Jiang et al., 2018)^,^ and 19 studies reported Observed Species (Prehn-Kristensen et al., 2018; Pulikkan et al., 2018; Coretti et al., 2018; Ding et al., 2020; Chen et al., 2020; Wan et al., 2022; Ye et al., 2021; Chen et al., 2021; Deng et al., 2022; Chiappori et al., 2022; He et al., 2023; Bundgaard-Nielsen et al., 2023; Wang et al., 2023; Zhao et al., 2023; Xu and Zhang, 2023; Bhusri et al., 2025; Szopinska-Tokov et al., 2020; Steckler et al., 2024; Boonchooduang et al., 2025). No significant differences were observed. The funnel plot in Supplementary Figure S4 indicated no signs of publication bias.

### 3.5 Beta diversity

Beta diversity assessments revealed significant compositional differences in gut microbiota between NDD patients and healthy controls, with distinct disorder-specific patterns. The most consistent findings emerged in ASD, where 16 of 33 studies (48.5%) reported significant differences using various diversity metrics (Kang et al., 2013; Strati et al., 2017; Pulikkan et al., 2018; Coretti et al., 2018; Zou et al., 2020; Chen et al., 2020; Wan et al., 2022; Ye et al., 2021; Huang et al., 2021; Deng et al., 2022; He et al., 2023; Bundgaard-Nielsen et al., 2023; Wang et al., 2023; Zhao et al., 2023; Pang et al., 2023; Li et al., 2023), while 3 studies (9.1%) showed non-significant results (Chen et al., 2021; Ding et al., 2021; Chen et al., 2022) and 4 studies (12.1%) exhibited metric-dependent variations (Zhang et al., 2018; Ma et al., 2019; Ding et al., 2020; Chen et al., 2022). For ADHD, only 2 of 10 studies (20%) identified significant differences (Prehn-Kristensen et al., 2018; Szopinska-Tokov et al., 2020) compared to 5 negative reports (50%) (Jiang et al., 2018; Wang et al., 2020; Richarte et al., 2021; Steckler et al., 2024; Panpetch et al., 2024), with 1 study (10%) showing inconsistent results (Boonchooduang et al., 2025). Preliminary evidence from 2 studies suggested microbial alterations in TD (Wang et al., 2022; Bao et al., 2023). These disorder-specific patterns, potentially influenced by methodological heterogeneity (diversity metrics, analytical approaches) and cohort characteristics, underscore the importance of considering NDD subtypes when evaluating gut microbiome perturbations. Detailed methodology and results for beta-diversity analyses are provided in Supplementary Table S7.

### 3.6 Microbial composition

**Figure 3:**
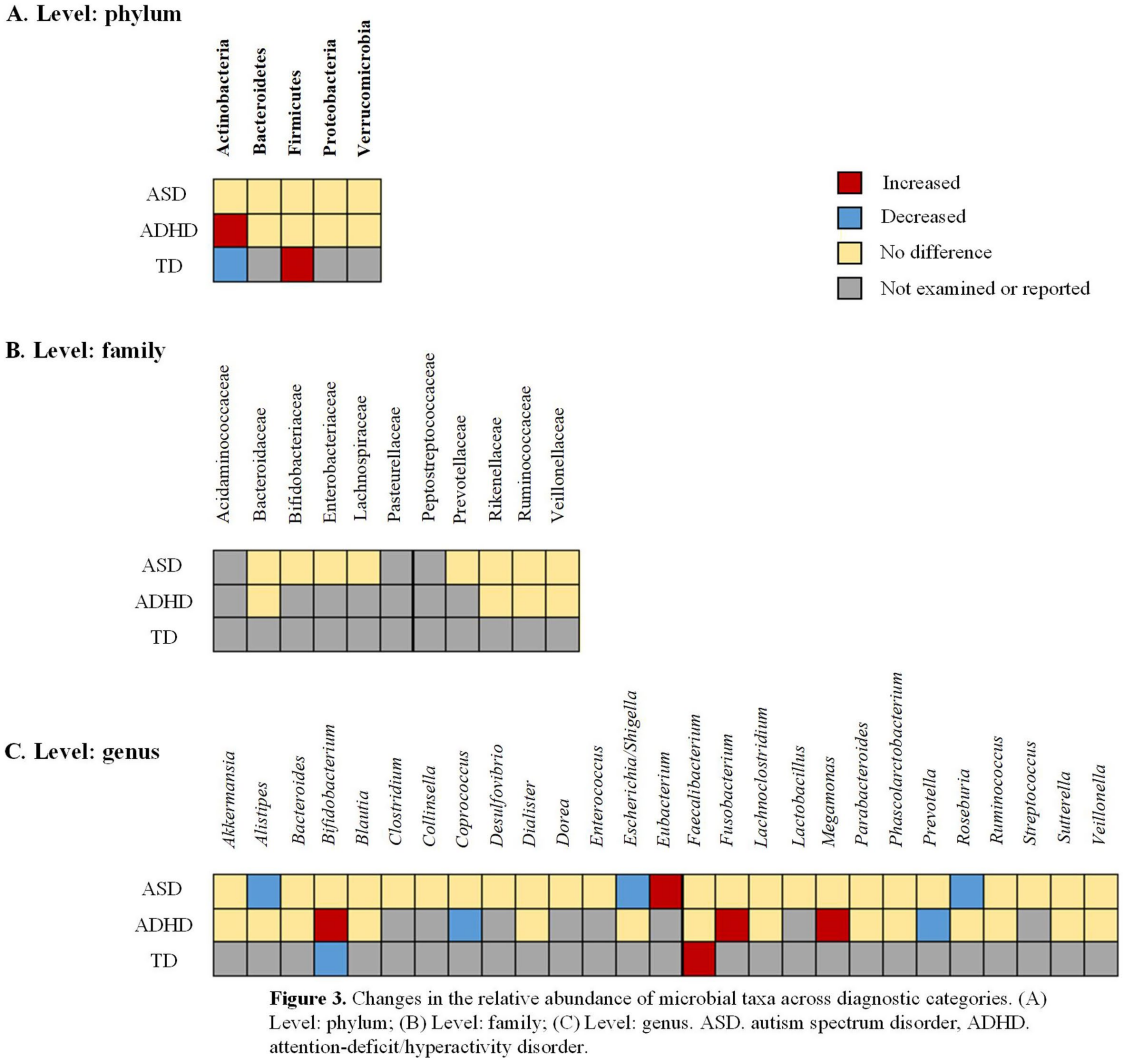
Changes in the relative abundance of microbial taxa across diagnostic categories. **(A)** Level: phylum; **(B)** Level: family; **(C)** Level: genus. ASD, autism spectrum disorder; ADHD, attention-deficit/hyperactivity disorder.

Relative abundance of gut microbiota was evaluated in 28 of 45 studies. Combined effect sizes across phylum, family, and genus categories are presented in Supplementary Table S4. Figure 3 illustrates gut microbiota changes in ADHD, ASD, and TD patients compared to controls, revealing considerable within-disorder variability that merits further investigation.

**Figure 4:**
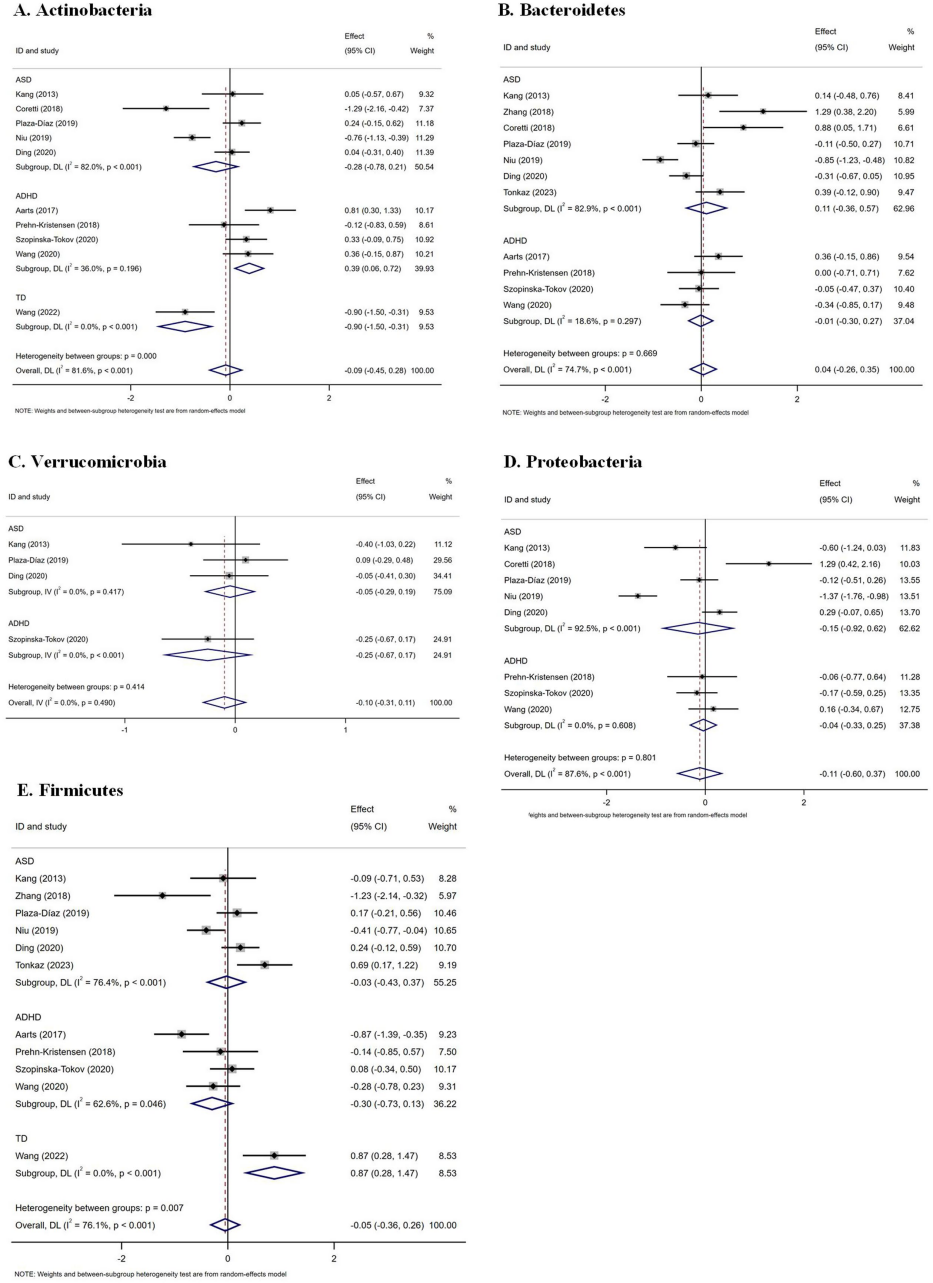
Forest plots of gut microbiota at the phylum level in patients with neurodevelopmental disorders compared with healthy controls. **(A)** Actinobacteria; **(B)** Bacteroidetes; **(C)** Verrucomicrobia; **(D)** Proteobacteria; **(E)** Firmicutes. ASD, autism spectrum disorder; ADHD, attention-deficit/hyperactivity disorder; TD, tic disorder.

At the phylum level, analysis of 10 studies investigating Actinobacteria revealed no significant overall difference between NDD patients and controls (Kang et al., 2013; Prehn-Kristensen et al., 2018; Aarts et al., 2017; Wang et al., 2022; Coretti et al., 2018; Plaza-Diaz et al., 2019; Niu et al., 2019; Ding et al., 2020; Szopinska-Tokov et al., 2020; Wang et al., 2020). However, subgroup analysis demonstrated a significant increase in Actinobacteria in ADHD (SMD = 0.39; 95% CI: 0.06 to 0.72; *p* = 0.020; *I*^2^ = 36.0%), contrasting with significantly lower levels in TD (SMD = −0.90; 95% CI: −1.50 to −0.31; *p* = 0.003; *I*^2^ = 0). Analysis of 11 studies each for Bacteroidetes (Kang et al., 2013; Prehn-Kristensen et al., 2018; Aarts et al., 2017; Zhang et al., 2018; Coretti et al., 2018; Plaza-Diaz et al., 2019; Niu et al., 2019; Ding et al., 2020; Yitik Tonkaz et al., 2023; Szopinska-Tokov et al., 2020; Wang et al., 2020) and Firmicutes (Kang et al., 2013; Prehn-Kristensen et al., 2018; Aarts et al., 2017; Wang et al., 2022; Zhang et al., 2018; Plaza-Diaz et al., 2019; Niu et al., 2019; Ding et al., 2020; Yitik Tonkaz et al., 2023; Szopinska-Tokov et al., 2020; Wang et al., 2020) revealed no significant overall differences, though Firmicutes was significantly elevated in the TD subgroup (SMD = 0.87; 95% CI: 0.28 to 1.47; *p* = 0.004; *I*^2^ = 0). No significant differences were observed for Proteobacteria and Verrucomicrobia (Figure 4).

**Figure 5:**
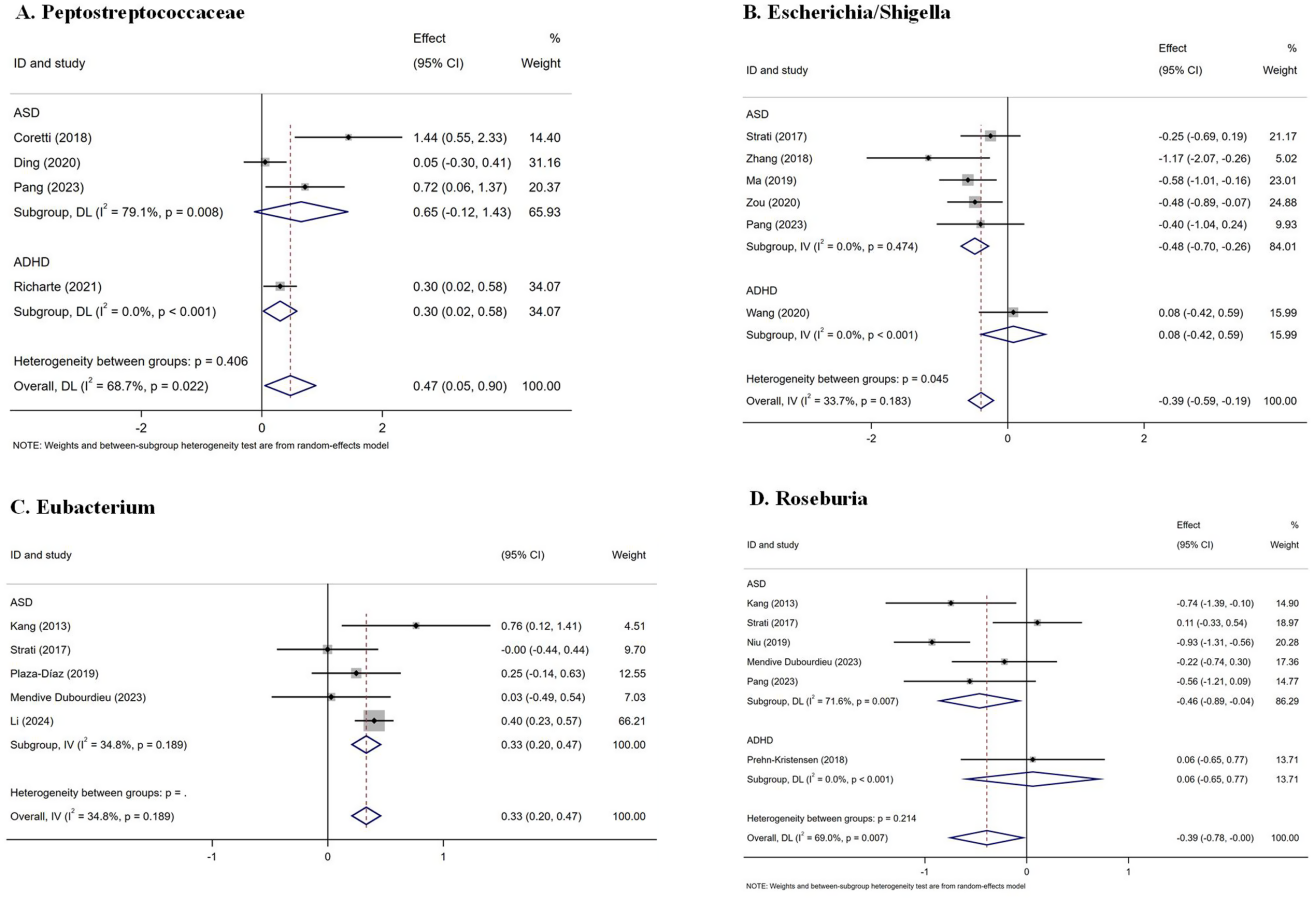
Forest plots of gut microbiota at the family and genus level in patients with neurodevelopmental disorders compared with healthy controls. **(A)** Peptostreptococcaceae; **(B)** Escherichia/Shigella; **(C)** Eubacterium; **(D)** Roseburia. ASD, autism spectrum disorder; ADHD, attention-deficit/hyperactivity disorder.

At the family level, a preliminary meta-analysis of only four studies assessing Peptostreptococcaceae suggested a significant increase in patients (SMD = 0.47; 95% CI: 0.05 to 0.90; *p* = 0.028; *I*^2^ = 68.7%), with subgroup analysis indicating a potential elevation in ADHD (SMD = 0.30; 95% CI: 0.02 to 0.58; *p* = 0.033; *I*^2^ = 0). However, this finding should be interpreted with caution due to the limited number of contributing studies (Figure 5).

At the genus level, analysis of 6 studies demonstrated a significant decrease in *Escherichia/Shigella* in NDD patients (SMD = −0.39; 95% CI: −0.59 to −0.19; *p* < 0.001; *I*^2^ = 33.7%), particularly pronounced in ASD (SMD = −0.48; 95% CI: −0.70 to −0.26; *p* < 0.001; *I*^2^ = 0). Conversely, 5 studies revealed a significant increase in *Eubacterium* (SMD = 0.33; 95% CI: 0.20 to 0.47; *p* < 0.001; *I*^2^ = 34.8%) (Kang et al., 2013; Strati et al., 2017; Plaza-Diaz et al., 2019; Mendive Dubourdieu and Guerendiain, 2023; Li et al., 2023). Analysis of 6 studies investigating *Roseburia* showed a significant decrease in patients (SMD = −0.39; 95% CI: −0.78 to 0; *p* = 0.049; *I*^2^ = 69.0%) (Kang et al., 2013; Prehn-Kristensen et al., 2018; Strati et al., 2017; Niu et al., 2019; Mendive Dubourdieu and Guerendiain, 2023; Pang et al., 2023), with significant reduction in ASD (SMD = −0.46; 95% CI: −0.89 to −0.04; *p* = 0.033; *I*^2^ = 71.6%). Supplementary material analysis revealed extensive heterogeneity in study-level findings across ADHD, ASD, and TD (Supplementary Figure S5).

## 4 Discussion

This systematic review and meta-analysis reveal significant alterations in gut microbiota composition among individuals with NDDs, reinforcing the crucial role of the gut-brain axis in these disorders. The substantial variability in study designs and demographic characteristics reflects the complex involvement of gut microbiota in NDD pathogenesis. Our findings provide comprehensive insights into microbial diversity and structural changes across ASD, ADHD, and TD. Despite considerable methodological heterogeneity, we identified consistent patterns that merit further investigation.

In our meta-analysis of alpha diversity indices, we observed no significant differences between NDD patients and healthy controls for the most commonly used indices, such as Chao1, Shannon, Simpson, and ACE. This suggests that global gut microbiota diversity, as measured by alpha diversity indices, may not be substantially altered in NDD patients. However, our findings indicate heterogeneous results within different NDD subtypes, such as ASD, ADHD, and TD. The gut microbiome’s alpha diversity in individuals with ASD shows inconsistent patterns when compared to healthy controls. Some studies reported higher richness and diversity in ASD patients (Zhai et al., 2019; Kang et al., 2018), while others found lower diversity (Wu et al., 2020; Liu et al., 2019), with some showing no significant difference (Strati et al., 2017; Carissimi et al., 2019). Similarly, ADHD studies demonstrated both decreased diversity (Prehn-Kristensen et al., 2018; Steckler et al., 2024), and no significant difference (Bundgaard-Nielsen et al., 2023; Wan et al., 2020; Bundgaard-Nielsen et al., 2020). Our study, with its large sample size and broader range of NDDs, did not find consistent evidence of reduced diversity, suggesting that alpha diversity may not be a reliable biomarker across all NDDs. It is noteworthy that certain alpha diversity indices, such as ACE, exhibited significant heterogeneity. This variability may be attributed to specific study factors (e.g., patient characteristics or differences in microbiota analysis methodologies), which could influence the results.

The meta-analysis of beta diversity showed more varied results. Seven studies observed no notable differences between patients and controls, while others identified distinct microbial clustering in individuals with ASD, ADHD, and TD, suggesting that specific NDDs may be associated with unique gut microbiota profiles. These findings are consistent with previous studies that reported altered beta diversity in ASD, ADHD, and TD (Bundgaard-Nielsen et al., 2023; Steckler et al., 2024; Yap et al., 2021). However, the assessment methods for beta diversity (such as principal coordinate analysis (PCoA) or other distance metrics) may contribute to variability in the consistency of these results. Our findings also suggest that diagnostic categories may exert a greater influence on microbiota composition than a general NDD diagnosis. For instance, in ASD and TD, the microbiota differences between patients and controls were more pronounced, possibly reflecting more consistent and robust identification of microbiota dysbiosis in these disorders (Chen et al., 2024; Xie et al., 2022). In contrast, the results for ADHD were less clear and more variable, possibly due to greater heterogeneity in their pathophysiology and microbiota composition.

This meta-analysis investigated gut microbiome abundance at the phylum, family, and genus levels and found no notable variations in Actinobacteria, Bacteroidetes, Firmicutes, Proteobacteria, or Verrucomicrobia between individuals with NDDs and healthy controls at the phylum level. However, subgroup analysis within NDDs revealed a significant increase in Actinobacteria at the genus level in patients with ADHD, while a significant decrease was observed in patients with TD (Aarts et al., 2017; Wang et al., 2022). The role of Actinobacteria may vary among different types of NDDs. The increase in Actinobacteria in ADHD patients may relate to neuroimmune dysregulation, which leads to behavioral abnormalities. Neuroimmune dysregulation could affect neuroinflammation and neurotransmitter signaling, thereby influencing gut-brain axis communication pathways (Chen et al., 2021; Abdel-Haq et al., 2019; Zhou et al., 2022). Our analysis also found a significant increase in Firmicutes in patients with TD. Short-chain fatty acids (SCFAs) modulate neuroinflammation, support brain function, and promote gut health. The increase in Firmicutes may influence the gut-brain axis through SCFA modulation, leading to corresponding clinical manifestations (Den Besten et al., 2013; Boukthir et al., 2010; De Magistris et al., 2010). No notable variations occurred in Bacteroidetes, Proteobacteria, and Verrucomicrobia between individuals with NDDs and healthy controls, implying that these particular phyla might exhibit greater stability across NDDs.

At the family level, our analysis revealed a significant elevation in Peptostreptococcaceae abundance among individuals with NDDs, with the most pronounced increase observed in ADHD (Jiang et al., 2018; Richarte et al., 2021). This finding corroborates growing evidence suggesting gut microbial dysbiosis as a potential contributor to neuropsychiatric pathogenesis (Szopinska-Tokov et al., 2020). Furthermore, Ruminococcaceae levels demonstrated a specific association with core symptoms of inattention, highlighting potential microbiota-behavior relationships in NDD (Tang et al., 2022). An elevated abundance of Peptostreptococcaceae may adversely affect the nervous system through immune-inflammatory pathways. Specifically, these bacteria can initiate pro-inflammatory responses that stimulate intestinal epithelial cells to release cytokines, including IL-6 and TNF-*α*. The resulting local inflammation weakens intestinal barrier function, permitting microbial products such as lipopolysaccharides to enter systemic circulation and induce low-grade systemic inflammation. This inflammatory state can traverse the blood–brain barrier, activate microglia, and promote neuroinflammation, ultimately impairing neuronal function and synaptic plasticity. These processes are considered integral to NDD pathophysiology (Palanivelu et al., 2024; Efremova et al., 2024). Moreover, certain clostridial species produce phenolic compounds such as phenol and p-cresol, which demonstrate neurotoxicity and may disrupt dopamine and norepinephrine metabolism in ADHD (Zhang et al., 2025). The between-study heterogeneity in the overall analysis may reflect diagnostic heterogeneity across NDDs or methodological variations in microbiota assessment. These results suggest Peptostreptococcaceae as a potential microbial marker warranting further investigation in NDDs, particularly regarding its role in modulating gut-brain communication through metabolic and immune pathways.

At the genus level, our analysis showed a notable decrease in the abundance of *Escherichia/Shigella* in patients compared to the control group, aligning with the results of Zou et al. (2020) regarding gut dysbiosis in ASD patients. The decrease of *Escherichia/Shigella* in the gut of NDD patients, especially ASD patients, may relate to decreased resistance to pathogenic microorganisms (Wang et al., 2020). Strati et al. (2017) also reported that in ASD patients, the abundance of *Escherichia/Shigella* was associated with gastrointestinal symptoms. Our study suggests that the significant decrease in the abundance of *Escherichia/Shigella* in ASD patients supports the hypothesis that alterations in these genera may contribute to the development of ASD. In contrast, we found a significant increase in the abundance of *Eubacterium* in patients, which aligns with the findings of Mendive Dubourdieu and Guerendiain (2023) *Eubacterium*, a key producer of SCFAs particularly butyrate, plays a crucial role in dietary fiber fermentation. While butyrate contributes to gut homeostasis by energizing colonocytes, strengthening the intestinal barrier, and exerting anti-inflammatory effects, elevated levels may exert paradoxical neurobehavioral effects. Evidence suggests that excess butyrate from specific microbial sources can influence neurodevelopment through epigenetic regulation of gene expression or direct interference with mitochondrial function (Dinan and Cryan, 2017; Srikantha and Mohajeri, 2019). A recent study using ASD patient-derived intestinal organoids demonstrated that metabolites from specific *Eubacterium* strains modulate neuronal activity, providing direct evidence for their role in gut-brain communication (Li et al., 2023). Consequently, the increased abundance of *Eubacterium* observed in NDDs may represent an adaptive response to dietary or gastrointestinal alterations, potentially influencing neuroinflammatory processes through SCFA-mediated pathways. Notably, our study demonstrated a marked decrease in *Roseburia*, consistent with reports by Kang et al. (2013). The decreased abundance of *Roseburia* leads to reduced butyrate levels in the gut, which may compromise intestinal barrier integrity. Insufficient butyrate supply impairs colonocyte energy metabolism, downregulates tight junction protein expression, and increases intestinal permeability, thereby facilitating the entry of neuroactive or pro-inflammatory substances into systemic circulation. Concurrently, diminished anti-inflammatory activity due to butyrate deficiency disinhibits pro-inflammatory signaling pathways such as NF-κB, potentially amplifying neuroinflammatory responses in the central nervous system (Niu et al., 2019; Guevara-Ramirez et al., 2025). Thus, the depletion of Roseburia likely represents a key factor driving the pathophysiology of NDDs. These findings emphasize the important role of gut microbiota composition in neurodevelopmental disorders and suggest that specific microbial taxa could serve as potential therapeutic targets for intervention.

The consistent microbial alterations identified in our study, particularly the enrichment of Peptostreptococcaceae and depletion of butyrate-producing *Roseburia*, provide a compelling rationale for microbiome-targeted interventions in NDDs. Probiotic supplementation with specific strains has demonstrated efficacy in improving both gastrointestinal and behavioral symptoms in children with ASD and ADHD (Tan et al., 2021; Novau-Ferre et al., 2025). Furthermore, prebiotic interventions, such as galacto-oligosaccharides, can modulate gut microbiota composition and improve attentional set-shifting performance (Gronier et al., 2018). Dietary strategies, including Mediterranean-style diets rich in fermentable fibers, may also help restore microbial balance and support gut-brain axis function (Park et al., 2024; Young et al., 2022). However, future interventions should account for the substantial heterogeneity observed across NDDs by adopting personalized approaches based on individual microbial profiles and should be validated through larger, well-designed clinical trials to establish optimal formulations and treatment durations.

This meta-analysis has several limitations. First, an substantial imbalance exists in the distribution of studies across different NDDs. Research on ASD constitutes the majority of included studies, while studies focusing on ADHD and particularly TD remain limited. This skewed distribution may compromise the generalizability of our findings across the entire spectrum of NDDs. Secondly, probiotics and antibiotics can significantly affect microbiota composition, but some studies did not report the use of these agents. Lastly, the limited number of studies for certain outcomes restricted both the precision of our estimates and the exploration of heterogeneity sources through subgroup analyses, highlighting the need for larger cohorts in future research.

## 5 Conclusion

In summary, this meta-analysis demonstrates significant alterations in the gut microbiota of individuals with NDDs, with distinct microbial profiles emerging across different disorder subtypes. While patients with NDDs showed no significant differences in alpha diversity compared to healthy controls, we identified substantial variations in beta diversity and microbial composition at multiple taxonomic levels.

The consistent pattern of dysbiosis, characterized by a trend toward increased Peptostreptococcaceae based on preliminary evidence alongside decreased *Escherichia/Shigella* and *Roseburia*, suggests these taxa may serve as potential microbial markers for NDDs. Microbiome-targeted interventions, including probiotic supplementation and dietary modifications, represent promising approaches for alleviating clinical symptoms in affected individuals. However, future large-scale, longitudinal studies are necessary to elucidate the causal relationships between gut microbiota and NDD pathophysiology and to develop personalized therapeutic strategies.

## Data Availability

The original contributions presented in the study are included in the article/Supplementary material, further inquiries can be directed to the corresponding author/s.

## Footnotes

1
http://www.math.hkbu.edu.hk/~tongt/papers/median2mean.html

## References

[ref1] Aarts E. Ederveen T. H. A. Naaijen J. Zwiers M. P. Boekhorst J. Timmerman H. M. . (2017). Gut microbiome in ADHD and its relation to neural reward anticipation. PLoS One 12:e0183509. doi: 10.1371/journal.pone.0183509, 28863139 PMC5581161

[ref2] Abdel-Haq R. Schlachetzki J. C. M. Glass C. K. Mazmanian S. K. (2019). Microbiome-microglia connections via the gut-brain axis. J. Exp. Med. 216, 41–59. doi: 10.1084/jem.20180794, 30385457 PMC6314531

[ref3] Abhishek F. Gugnani J. S. Kaur H. Damera A. R. Mane R. Sekhri A. . (2024). Dietary interventions and supplements for managing attention-deficit/hyperactivity disorder (ADHD): a systematic review of efficacy and recommendations. Cureus 16:e69804. doi: 10.7759/cureus.69804, 39429382 PMC11491108

[ref4] Altaib H. Nakamura K. Abe M. Badr Y. Yanase E. Nomura I. . (2021). Differences in the concentration of the fecal neurotransmitters GABA and glutamate are associated with microbial composition among healthy human subjects. Microorganisms 9:378. doi: 10.3390/microorganisms9020378, 33668550 PMC7918917

[ref5] American Psychiatric Association (2013). Diagnostic and statistical manual of mental disorders: DSM-5. 5th Edn. Washington, DC: American Psychiatric Association.

[ref6] Aresti-Sanz J. Schwalbe M. Pereira R. R. Permentier H. El Aidy S. (2021). Stability of methylphenidate under various pH conditions in the presence or absence of gut microbiota. Pharmaceuticals (Basel) 14:733. doi: 10.3390/ph14080733, 34451830 PMC8398889

[ref7] Bao C. Wei M. Pan H. Wen M. Liu Z. Xu Y. . (2023). A preliminary study for the clinical effect of one combinational physiotherapy and its potential influence on gut microbial composition in children with Tourette syndrome. Front. Nutr. 10:1184311. doi: 10.3389/fnut.2023.1184311, 37781119 PMC10541309

[ref8] Bezawada N. Phang T. H. Hold G. L. Hansen R. (2020). Autism spectrum disorder and the gut microbiota in children: a systematic review. Ann. Nutr. Metab. 76, 16–29. doi: 10.1159/000505363 31982866

[ref9] Bhusri B. Sutheeworapong S. Kittichotirat W. Kusonmano K. Thammarongtham C. Lertampaiporn S. . (2025). Characterization of gut microbiota on gender and age groups bias in Thai patients with autism spectrum disorder. Sci. Rep. 15:2587. doi: 10.1038/s41598-025-86740-2, 39833480 PMC11747245

[ref10] Boonchooduang N. Louthrenoo O. Likhitweerawong N. Kunasol C. Thonusin C. Sriwichaiin S. . (2025). Impact of psychostimulants on microbiota and short-chain fatty acids alterations in children with attention-deficit/hyperactivity disorder. Sci. Rep. 15:3034. doi: 10.1038/s41598-025-87546-y, 39856212 PMC11759945

[ref11] Borre Y. E. O'keeffe G. W. Clarke G. Stanton C. Dinan T. G. Cryan J. F. (2014). Microbiota and neurodevelopmental windows: implications for brain disorders. Trends Mol. Med. 20, 509–518. doi: 10.1016/j.molmed.2014.05.002, 24956966

[ref12] Boukthir S. Matoussi N. Belhadj A. Mammou S. Dlala S. B. Helayem M. . (2010). Abnormal intestinal permeability in children with autism. Tunis. Med. 88, 685–686. 20812190

[ref13] Bull-Larsen S. Mohajeri M. H. (2019). The potential influence of the bacterial microbiome on the development and progression of ADHD. Nutrients 11:805. doi: 10.3390/nu11112805, 31744191 PMC6893446

[ref14] Bundgaard-Nielsen C. Knudsen J. Leutscher P. D. C. Lauritsen M. B. Nyegaard M. Hagstrom S. . (2020). Gut microbiota profiles of autism spectrum disorder and attention deficit/hyperactivity disorder: a systematic literature review. Gut Microbes 11, 1172–1187. doi: 10.1080/19490976.2020.1748258, 32329656 PMC7524304

[ref15] Bundgaard-Nielsen C. Lauritsen M. B. Knudsen J. K. Rold L. S. Larsen M. H. Hindersson P. . (2023). Children and adolescents with attention deficit hyperactivity disorder and autism spectrum disorder share distinct microbiota compositions. Gut Microbes 15:2211923. doi: 10.1080/19490976.2023.2211923, 37199526 PMC10197996

[ref16] Cao X. Liu K. Liu J. Liu Y. W. Xu L. Wang H. . (2021). Dysbiotic gut microbiota and dysregulation of cytokine profile in children and teens with autism Spectrum disorder. Front. Neurosci. 15:635925. doi: 10.3389/fnins.2021.635925, 33642989 PMC7902875

[ref17] Carissimi C. Laudadio I. Palone F. Fulci V. Cesi V. Cardona F. . (2019). Functional analysis of gut microbiota and immunoinflammation in children with autism spectrum disorders. Dig. Liver Dis. 51, 1366–1374. doi: 10.1016/j.dld.2019.06.006, 31320306

[ref18] Chen Y. Fang H. Li C. Wu G. Xu T. Yang X. . (2020). Gut bacteria shared by children and their mothers associate with developmental level and social deficits in autism spectrum disorder. mSphere 5:1044. doi: 10.1128/mSphere.01044-20, 33268567 PMC7716279

[ref19] Chen H. D. Li L. Yu F. Ma Z. S. (2024). A comprehensive diversity analysis on the gut microbiomes of ASD patients: from alpha, beta to gamma diversities. FEMS Microbiol. Lett. 371:14. doi: 10.1093/femsle/fnae014, 38419294

[ref20] Chen Y. C. Lin H. Y. Chien Y. Tung Y. H. Ni Y. H. Gau S. S. (2022). Altered gut microbiota correlates with behavioral problems but not gastrointestinal symptoms in individuals with autism. Brain Behav. Immun. 106, 161–178. doi: 10.1016/j.bbi.2022.08.015, 36058421

[ref21] Chen Z. Shi K. Liu X. Dai Y. Liu Y. Zhang L. . (2021). Gut microbial profile is associated with the severity of social impairment and IQ performance in children with autism Spectrum disorder. Front. Psych. 12:789864. doi: 10.3389/fpsyt.2021.789864, 34975585 PMC8718873

[ref22] Chen Y. Xu J. Chen Y. (2021). Regulation of neurotransmitters by the gut microbiota and effects on cognition in neurological disorders. Nutrients 13:99. doi: 10.3390/nu13062099, 34205336 PMC8234057

[ref23] Chernikova M. A. Flores G. D. Kilroy E. Labus J. S. Mayer E. A. Aziz-Zadeh L. (2021). The brain-gut-microbiome system: pathways and implications for autism Spectrum disorder. Nutrients 13:497. doi: 10.3390/nu13124497, 34960049 PMC8704412

[ref24] Chiappori F. Cupaioli F. A. Consiglio A. Di Nanni N. Mosca E. Licciulli V. F. . (2022). Analysis of Faecal microbiota and small ncRNAs in autism: detection of miRNAs and piRNAs with possible implications in host-gut microbiota cross-talk. Nutrients 14:1340. doi: 10.3390/nu14071340, 35405953 PMC9000903

[ref25] Clarke G. Stilling R. M. Kennedy P. J. Stanton C. Cryan J. F. Dinan T. G. (2014). Minireview: gut microbiota: the neglected endocrine organ. Mol. Endocrinol. 28, 1221–1238. doi: 10.1210/me.2014-1108, 24892638 PMC5414803

[ref26] Coretti L. Paparo L. Riccio M. P. Amato F. Cuomo M. Natale A. . (2018). Gut microbiota features in Young children with autism Spectrum disorders. Front. Microbiol. 9:3146. doi: 10.3389/fmicb.2018.03146, 30619212 PMC6305749

[ref27] Cryan J. F. O'riordan K. J. Cowan C. S. M. Sandhu K. V. Bastiaanssen T. F. S. Boehme M. . (2019). The microbiota-gut-brain Axis. Physiol. Rev. 99, 1877–2013. doi: 10.1152/physrev.00018.2018 31460832

[ref28] Dall’Aglio L. Muka T. Cecil C. A. M. Bramer W. M. Verbiest M. Nano J. . (2018). The role of epigenetic modifications in neurodevelopmental disorders: a systematic review. Neurosci. Biobehav. Rev. 94, 17–30. doi: 10.1016/j.neubiorev.2018.07.011, 30067938

[ref29] Dash S. Syed Y. A. Khan M. R. (2022). Understanding the role of the gut microbiome in brain development and its association with neurodevelopmental psychiatric disorders. Front. Cell Dev. Biol. 10:880544. doi: 10.3389/fcell.2022.880544, 35493075 PMC9048050

[ref30] De Jong S. Newhouse S. J. Patel H. Lee S. Dempster D. Curtis C. . (2016). Immune signatures and disorder-specific patterns in a cross-disorder gene expression analysis. Br. J. Psychiatry 209, 202–208. doi: 10.1192/bjp.bp.115.175471, 27151072 PMC5007452

[ref31] De Magistris L. Familiari V. Pascotto A. Sapone A. Frolli A. Iardino P. . (2010). Alterations of the intestinal barrier in patients with autism spectrum disorders and in their first-degree relatives. J. Pediatr. Gastroenterol. Nutr. 51, 418–424. doi: 10.1097/MPG.0b013e3181dcc4a5, 20683204

[ref32] De Theije C. G. Wopereis H. Ramadan M. Van Eijndthoven T. Lambert J. Knol J. . (2014). Altered gut microbiota and activity in a murine model of autism spectrum disorders. Brain Behav. Immun. 37, 197–206. doi: 10.1016/j.bbi.2013.12.005, 24333160

[ref33] Den Besten G. Van Eunen K. Groen A. K. Venema K. Reijngoud D. J. Bakker B. M. (2013). The role of short-chain fatty acids in the interplay between diet, gut microbiota, and host energy metabolism. J. Lipid Res. 54, 2325–2340. doi: 10.1194/jlr.R036012, 23821742 PMC3735932

[ref34] Deng W. Wang S. Li F. Wang F. Xing Y. P. Li Y. . (2022). Gastrointestinal symptoms have a minor impact on autism spectrum disorder and associations with gut microbiota and short-chain fatty acids. Front. Microbiol. 13:1000419. doi: 10.3389/fmicb.2022.1000419, 36274684 PMC9585932

[ref35] Dinan T. G. Cryan J. F. (2017). Gut instincts: microbiota as a key regulator of brain development, ageing and neurodegeneration. J. Physiol. 595, 489–503. doi: 10.1113/JP273106, 27641441 PMC5233671

[ref36] Ding X. Xu Y. Zhang X. Zhang L. Duan G. Song C. . (2020). Gut microbiota changes in patients with autism spectrum disorders. J. Psychiatr. Res. 129, 149–159. doi: 10.1016/j.jpsychires.2020.06.032, 32912596

[ref37] Ding H. Yi X. Zhang X. Wang H. Liu H. Mou W. W. (2021). Imbalance in the gut microbiota of children with autism Spectrum disorders. Front. Cell. Infect. Microbiol. 11:572752. doi: 10.3389/fcimb.2021.572752, 34790583 PMC8591234

[ref38] Efremova I. Maslennikov R. Kudryavtseva A. Avdeeva A. Krasnov G. Diatroptov M. . (2024). Gut microbiota and cytokine profile in cirrhosis. J. Clin. Transl. Hepatol. 12, 689–700. doi: 10.14218/JCTH.2024.00090, 39130620 PMC11310756

[ref39] Geng J. Liu C. Xu J. Wang X. Li X. (2023). Potential relationship between Tourette syndrome and gut microbiome. J. Pediatr. 99, 11–16. doi: 10.1016/j.jped.2022.06.002, 35914739 PMC9875241

[ref40] Goncalves C. L. Doifode T. Rezende V. L. Costa M. A. Rhoads J. M. Soutullo C. A. (2024). The many faces of microbiota-gut-brain axis in autism spectrum disorder. Life Sci. 337:122357. doi: 10.1016/j.lfs.2023.122357, 38123016

[ref41] Gronier B. Savignac H. M. Di Miceli M. Idriss S. M. Tzortzis G. Anthony D. . (2018). Increased cortical neuronal responses to NMDA and improved attentional set-shifting performance in rats following prebiotic (B-GOS((R))) ingestion. Eur. Neuropsychopharmacol. 28, 211–224. doi: 10.1016/j.euroneuro.2017.11.001, 29174530 PMC5857269

[ref42] Guevara-Ramirez P. Tamayo-Trujillo R. Ruiz-Pozo V. A. Cadena-Ullauri S. Paz-Cruz E. Zambrano A. K. (2025). Mechanistic links between gut Dysbiosis, insulin resistance, and autism Spectrum disorder. Int. J. Mol. Sci. 26:537. doi: 10.3390/ijms26136537, 40650313 PMC12249569

[ref43] He J. Gong X. Hu B. Lin L. Lin X. Gong W. . (2023). Altered gut microbiota and short-chain fatty acids in Chinese children with constipated autism Spectrum disorder. Sci. Rep. 13:19103. doi: 10.1038/s41598-023-46566-2, 37925571 PMC10625580

[ref44] Huang M. Liu J. Liu K. Chen J. Wei Z. Feng Z. . (2021). Microbiome-specific statistical modeling identifies interplay between gastrointestinal microbiome and neurobehavioral outcomes in patients with autism: a case control study. Front. Psych. 12:682454. doi: 10.3389/fpsyt.2021.682454, 34744810 PMC8563626

[ref45] Jiang H. Y. Zhou Y. Y. Zhou G. L. Li Y. C. Yuan J. Li X. H. . (2018). Gut microbiota profiles in treatment-naïve children with attention deficit hyperactivity disorder. Behav. Brain Res. 347, 408–413. doi: 10.1016/j.bbr.2018.03.036, 29580894

[ref46] Kanaan A. S. Gerasch S. Garcia-Garcia I. Lampe L. Pampel A. Anwander A. . (2017). Pathological glutamatergic neurotransmission in Gilles de la Tourette syndrome. Brain 140, 218–234. doi: 10.1093/brain/aww285 28007998

[ref47] Kang D. W. Ilhan Z. E. Isern N. G. Hoyt D. W. Howsmon D. P. Shaffer M. . (2018). Differences in fecal microbial metabolites and microbiota of children with autism spectrum disorders. Anaerobe 49, 121–131. doi: 10.1016/j.anaerobe.2017.12.007, 29274915

[ref48] Kang D. W. Park J. G. Ilhan Z. E. Wallstrom G. Labaer J. Adams J. B. . (2013). Reduced incidence of Prevotella and other fermenters in intestinal microflora of autistic children. PLoS One 8:e68322. doi: 10.1371/journal.pone.0068322, 23844187 PMC3700858

[ref49] Kovtun A. S. Averina O. V. Alekseeva M. G. Danilenko V. N. (2020). Antibiotic resistance genes in the gut microbiota of children with autistic Spectrum disorder as possible predictors of the disease. Microb. Drug Resist. 26, 1307–1320. doi: 10.1089/mdr.2019.0325, 31916894

[ref50] Li H. Guo W. Li S. Sun B. Li N. Xie D. . (2023). Alteration of the gut microbiota profile in children with autism spectrum disorder in China. Front. Microbiol. 14:1326870. doi: 10.3389/fmicb.2023.1326870, 38420215 PMC10899803

[ref51] Liu S. Li E. Sun Z. Fu D. Duan G. Jiang M. . (2019). Altered gut microbiota and short chain fatty acids in Chinese children with autism spectrum disorder. Sci. Rep. 9:287. doi: 10.1038/s41598-018-36430-z, 30670726 PMC6342986

[ref52] Lord C. Elsabbagh M. Baird G. Veenstra-Vanderweele J. (2018). Autism spectrum disorder. Lancet 392, 508–520. doi: 10.1016/S0140-6736(18)31129-2, 30078460 PMC7398158

[ref53] Lukens J. R. Eyo U. B. (2022). Microglia and neurodevelopmental disorders. Annu. Rev. Neurosci. 45, 425–445. doi: 10.1146/annurev-neuro-110920-023056, 35436413 PMC10449242

[ref54] Ma B. Liang J. Dai M. Wang J. Luo J. Zhang Z. . (2019). Altered gut microbiota in Chinese children with autism Spectrum disorders. Front. Cell. Infect. Microbiol. 9:40. doi: 10.3389/fcimb.2019.00040, 30895172 PMC6414714

[ref55] Martinez-Gonzalez A. E. Andreo-Martinez P. (2020). Prebiotics, probiotics and fecal microbiota transplantation in autism: a systematic review. Rev. Psiquiatr. Salud Ment. (Engl. Ed.) 13, 150–164. doi: 10.1016/j.rpsm.2020.06.002, 32684346

[ref56] Mendive Dubourdieu P. Guerendiain M. (2023). Understanding the link between gut microbiota, dietary intake, and nutritional status in children with autism and typical development. Front. Nutr. 10:1202948. doi: 10.3389/fnut.2023.1202948, 37545578 PMC10399235

[ref57] Morris-Rosendahl D. J. Crocq M. A. (2020). Neurodevelopmental disorders-the history and future of a diagnostic concept. Dialogues Clin. Neurosci. 22, 65–72. doi: 10.31887/DCNS.2020.22.1/macrocq, 32699506 PMC7365295

[ref58] Nikolaus S. Mamlins E. Antke C. Dabir M. Muller H. W. Giesel F. L. (2022). Boosted dopamine and blunted serotonin in Tourette syndrome - evidence from in vivo imaging studies. Rev. Neurosci. 33, 859–876. doi: 10.1515/revneuro-2022-0035, 35575756

[ref59] Niu M. Li Q. Zhang J. Wen F. Dang W. Duan G. . (2019). Characterization of intestinal microbiota and probiotics treatment in children with autism Spectrum disorders in China. Front. Neurol. 10:1084. doi: 10.3389/fneur.2019.01084, 31749754 PMC6848227

[ref60] Novau-Ferre N. Papandreou C. Rojo-Marticella M. Canals-Sans J. Bullo M. (2025). Gut microbiome differences in children with attention deficit hyperactivity disorder and autism Spectrum disorder and effects of probiotic supplementation: a randomized controlled trial. Res. Dev. Disabil. 161:105003. doi: 10.1016/j.ridd.2025.105003, 40184961

[ref61] Page M. J. Mckenzie J. E. Bossuyt P. M. Boutron I. Hoffmann T. C. Mulrow C. D. . (2021). The PRISMA 2020 statement: an updated guideline for reporting systematic reviews. BMJ 372:n71. doi: 10.1136/bmj.n71, 33782057 PMC8005924

[ref62] Palanivelu L. Chen Y. Y. Chang C. J. Liang Y. W. Tseng H. Y. Li S. J. . (2024). Investigating brain-gut microbiota dynamics and inflammatory processes in an autistic-like rat model using MRI biomarkers during childhood and adolescence. NeuroImage 302:120899. doi: 10.1016/j.neuroimage.2024.120899, 39461606

[ref63] Pang X. Zhang Q. Wang Y. Zhan Y. Guo M. Chen B. . (2023). Characteristics of the gut microbiota in young adults with autism spectrum disorder. J. Integr. Neurosci. 22:141. doi: 10.31083/j.jin2206141, 38176916

[ref64] Panpetch J. Kiatrungrit K. Tuntipopipat S. Tangphatsornruang S. Mhuantong W. Chongviriyaphan N. (2024). Gut microbiota and clinical manifestations in Thai pediatric patients with attention-deficit hyperactivity disorder. J. Pers. Med. 14:739. doi: 10.3390/jpm14070739, 39063993 PMC11277806

[ref65] Park G. Kadyan S. Hochuli N. Pollak J. Wang B. Salazar G. . (2024). A modified Mediterranean-style diet enhances brain function via specific gut-microbiome-brain mechanisms. Gut Microbes 16:2323752. doi: 10.1080/19490976.2024.2323752, 38444392 PMC10936641

[ref66] Plaza-Diaz J. Gomez-Fernandez A. Chueca N. Torre-Aguilar M. J. Gil A. Perez-Navero J. L. . (2019). Autism spectrum disorder (ASD) with and without mental regression is associated with changes in the fecal microbiota. Nutrients 11:337. doi: 10.3390/nu11020337, 30764497 PMC6412819

[ref67] Prehn-Kristensen A. Zimmermann A. Tittmann L. Lieb W. Schreiber S. Baving L. . (2018). Reduced microbiome alpha diversity in young patients with ADHD. PLoS One 13:e0200728. doi: 10.1371/journal.pone.0200728, 30001426 PMC6042771

[ref68] Pulikkan J. Maji A. Dhakan D. B. Saxena R. Mohan B. Anto M. M. . (2018). Gut microbial Dysbiosis in Indian children with autism Spectrum disorders. Microb. Ecol. 76, 1102–1114. doi: 10.1007/s00248-018-1176-2, 29564487

[ref69] Richarte V. Sanchez-Mora C. Corrales M. Fadeuilhe C. Vilar-Ribo L. Arribas L. . (2021). Gut microbiota signature in treatment-naive attention-deficit/hyperactivity disorder.Transl. Psychiatry 11:382. doi: 10.1038/s41398-021-01504-6, 34238926 PMC8266901

[ref70] Song W. Zhang M. Teng L. Wang Y. Zhu L. (2022). Prebiotics and probiotics for autism spectrum disorder: a systematic review and meta-analysis of controlled clinical trials. J. Med. Microbiol. 71:1510. doi: 10.1099/jmm.0.001510, 35438624

[ref71] Srikantha P. Mohajeri M. H. (2019). The possible role of the microbiota-gut-brain-Axis in autism Spectrum disorder. Int. J. Mol. Sci. 20:2115. doi: 10.3390/ijms20092115, 31035684 PMC6539237

[ref72] Steckler R. Magzal F. Kokot M. Walkowiak J. Tamir S. (2024). Disrupted gut harmony in attention-deficit/hyperactivity disorder: Dysbiosis and decreased short-chain fatty acids. Brain Behav Immun Health 40:100829. doi: 10.1016/j.bbih.2024.100829, 39184374 PMC11342906

[ref73] Strati F. Cavalieri D. Albanese D. De Felice C. Donati C. Hayek J. . (2017). New evidences on the altered gut microbiota in autism spectrum disorders. Microbiome 5:24. doi: 10.1186/s40168-017-0242-1, 28222761 PMC5320696

[ref74] Sun H. You Z. Jia L. Wang F. (2019). Autism spectrum disorder is associated with gut microbiota disorder in children. BMC Pediatr. 19:516. doi: 10.1186/s12887-019-1896-6, 31881951 PMC6933684

[ref75] Szopinska-Tokov J. Dam S. Naaijen J. Konstanti P. Rommelse N. Belzer C. . (2020). Investigating the gut microbiota composition of individuals with attention-deficit/hyperactivity disorder and association with symptoms. Microorganisms 8:406. doi: 10.3390/microorganisms8030406, 32183143 PMC7143990

[ref76] Tan Q. Orsso C. E. Deehan E. C. Kung J. Y. Tun H. M. Wine E. . (2021). Probiotics, prebiotics, synbiotics, and fecal microbiota transplantation in the treatment of behavioral symptoms of autism spectrum disorder: a systematic review. Autism Res. 14, 1820–1836. doi: 10.1002/aur.2560, 34173726

[ref77] Tang K. Hao W. Mo X. Chen Y. Guo X. He L. . (2022). Analysis of the therapeutic effect of Dimu Ningshen (TCM formula) on attention deficit hyperactivity disorder based on gut microbiota and serum metabolomics. BMC Complement. Med. Ther. 22:24. doi: 10.1186/s12906-022-03512-5, 35078472 PMC8790860

[ref78] Thapar A. Cooper M. Eyre O. Langley K. (2013). What have we learnt about the causes of ADHD? J. Child Psychol. Psychiatry 54, 3–16. doi: 10.1111/j.1469-7610.2012.02611.x, 22963644 PMC3572580

[ref79] Thapar A. Cooper M. Rutter M. (2017). Neurodevelopmental disorders. Lancet Psychiatry 4, 339–346. doi: 10.1016/S2215-0366(16)30376-5 27979720

[ref80] Wan L. Ge W. R. Zhang S. Sun Y. L. Wang B. Yang G. (2020). Case-control study of the effects of gut microbiota composition on neurotransmitter metabolic pathways in children with attention deficit hyperactivity disorder. Front. Neurosci. 14:127. doi: 10.3389/fnins.2020.00127, 32132899 PMC7040164

[ref81] Wan Y. Zuo T. Xu Z. Zhang F. Zhan H. Chan D. . (2022). Underdevelopment of the gut microbiota and bacteria species as non-invasive markers of prediction in children with autism spectrum disorder. Gut 71, 910–918. doi: 10.1136/gutjnl-2020-324015, 34312160

[ref82] Wang L. Christophersen C. T. Sorich M. J. Gerber J. P. Angley M. T. Conlon M. A. (2011). Low relative abundances of the mucolytic bacterium *Akkermansia muciniphila* and *Bifidobacterium* spp. in feces of children with autism. Appl. Environ. Microbiol. 77, 6718–6721. doi: 10.1128/AEM.05212-11, 21784919 PMC3187122

[ref83] Wang H. Liu S. Xie L. Wang J. (2023). Gut microbiota signature in children with autism spectrum disorder who suffered from chronic gastrointestinal symptoms. BMC Pediatr. 23:476. doi: 10.1186/s12887-023-04292-8, 37730588 PMC10510216

[ref84] Wang Y. Xu H. Jing M. Hu X. Wang J. Hua Y. (2022). Gut microbiome composition abnormalities determined using high-throughput sequencing in children with tic disorder. Front. Pediatr. 10:831944. doi: 10.3389/fped.2022.831944, 35601424 PMC9114666

[ref85] Wang L. J. Yang C. Y. Chou W. J. Lee M. J. Chou M. C. Kuo H. C. . (2020). Gut microbiota and dietary patterns in children with attention-deficit/hyperactivity disorder. Eur. Child Adolesc. Psychiatry 29, 287–297. doi: 10.1007/s00787-019-01352-2, 31119393

[ref86] Wu T. Wang H. Lu W. Zhai Q. Zhang Q. Yuan W. . (2020). Potential of gut microbiome for detection of autism spectrum disorder. Microb. Pathog. 149:104568. doi: 10.1016/j.micpath.2020.104568, 33096147

[ref87] Xi W. Gao X. Zhao H. Luo X. Li J. Tan X. . (2021). Depicting the composition of gut microbiota in children with tic disorders: an exploratory study. J. Child Psychol. Psychiatry 62, 1246–1254. doi: 10.1111/jcpp.13409, 33738808

[ref88] Xie X. Li L. Wu X. Hou F. Chen Y. Shi L. . (2022). Alteration of the fecal microbiota in Chinese children with autism spectrum disorder. Autism Res. 15, 996–1007. doi: 10.1002/aur.2718, 35403356

[ref89] Xu X. Z. Y. Zhang X. (2023). Autism spectrum disorder is related to increasing intestinal Prevotella that can be regulated by vitamin a. Iran. J. Psychiatry Behav. Sci. 17:126508. doi: 10.5812/ijpbs-126508

[ref90] Yap C. X. Henders A. K. Alvares G. A. Wood D. L. A. Krause L. Tyson G. W. . (2021). Autism-related dietary preferences mediate autism-gut microbiome associations. Cell 184, 5916–5931.e17. doi: 10.1016/j.cell.2021.10.015, 34767757

[ref91] Ye F. Gao X. Wang Z. Cao S. Liang G. He D. . (2021). Comparison of gut microbiota in autism spectrum disorders and neurotypical boys in China: a case-control study. Synth. Syst. Biotechnol. 6, 120–126. doi: 10.1016/j.synbio.2021.03.003, 34095558 PMC8163862

[ref92] Yitik Tonkaz G. Esin I. S. Turan B. Uslu H. Dursun O. B. (2023). Determinants of leaky gut and gut microbiota differences in children with autism Spectrum disorder and their siblings. J. Autism Dev. Disord. 53, 2703–2716. doi: 10.1007/s10803-022-05540-z, 35441922

[ref93] Young H. A. Freegard G. Benton D. (2022). Mediterranean diet, interoception and mental health: is it time to look beyond the gut-brain axis? Physiol. Behav. 257:113964. doi: 10.1016/j.physbeh.2022.113964, 36130628

[ref94] Zhai Q. Cen S. Jiang J. Zhao J. Zhang H. Chen W. (2019). Disturbance of trace element and gut microbiota profiles as indicators of autism spectrum disorder: a pilot study of Chinese children. Environ. Res. 171, 501–509. doi: 10.1016/j.envres.2019.01.060, 30743242

[ref95] Zhang M. Ma W. Zhang J. He Y. Wang J. (2018). Analysis of gut microbiota profiles and microbe-disease associations in children with autism spectrum disorders in China. Sci. Rep. 8:13981. doi: 10.1038/s41598-018-32219-2, 30228282 PMC6143520

[ref96] Zhang J. Yuan M. Liu Y. Zhong X. Wu J. Chen W. (2025). Bisphenol a exposure and neurodevelopmental disorders and problems in children under 12 years of age: a systematic review and meta-analysis. J. Hazard. Mater. 490:137731. doi: 10.1016/j.jhazmat.2025.137731, 40054188

[ref97] Zhao Y. Wang Y. Meng F. Chen X. Chang T. Huang H. . (2023). Altered gut microbiota as potential biomarkers for autism Spectrum disorder in early childhood. Neuroscience 523, 118–131. doi: 10.1016/j.neuroscience.2023.04.029, 37271221

[ref98] Zhou R. Qian S. Cho W. C. S. Zhou J. Jin C. Zhong Y. . (2022). Microbiota-microglia connections in age-related cognition decline. Aging Cell 21:e13599. doi: 10.1111/acel.13599, 35349746 PMC9124309

[ref99] Zou R. Xu F. Wang Y. Duan M. Guo M. Zhang Q. . (2020). Changes in the gut microbiota of children with autism spectrum disorder. Autism Res. 13, 1614–1625. doi: 10.1002/aur.2358, 32830918

