# Supplementary material for: Alterations in gut microbiota composition in neurodevelopmental disorders: a systematic review and meta-analysis
Source: Front Microbiol. 2025 Dec 9;16:1650212. doi: 10.3389/fmicb.2025.1650212 (PMC12723412; doi:10.3389/fmicb.2025.1650212)
Supplement: Supplementary file 6 [file Table_1.DOCX]

**Table S1.** Search Strategy for Each Electronic Database.

| **PubMed LIMIT: humans and English** |
| --- |
| 1. “Attention deficit disorder with hyperactivity”[MeSH] |
| 2. ADHD[Title/Abstract] OR ADDH[Title/Abstract] OR ADD[Title/Abstract] OR Attention[Title/Abstract] OR Inattention[Title/Abstract] OR Hyperactiv*[Title/Abstract] OR Impulsiv*[Title/Abstract] OR Hyperkinetic Disorder |
| 3. “Autism Spectrum Disorder”[MeSH] |
| 4. Autism Spectrum Disorder[Title/Abstract] OR Autistic Spectrum Disorders[Title/Abstract] OR Autistic Spectrum Disorder[Title/Abstract] OR Autistic Spectrum Disorders[Title/Abstract] OR Disorder, Autistic Spectrum[Title/Abstract] |
| 5. “Tourette syndrome” [MeSH] OR “Tic Disorders”[MeSH] |
| 6. Tourette syndrome[Title/Abstract] OR Tourette Disease[Title/Abstract] OR Tourette Disorder*[Title/Abstract] OR Tic Disorder*[Title/Abstract] OR Tourette’s Disorder [Title/Abstract] OR Syndrome, Tourette[Title/Abstract] |
| 7. Microbiota[MeSH] |
| 8. Microbiome[Title/Abstract] OR Ecosystem[Title/Abstract] OR Bacteria[Title/Abstract] OR Flora[Title/Abstract] OR Microflora[Title/Abstract] OR Dysbiosis[Title/Abstract] |
| 9. Gut[Title/Abstract] OR Gastrointestinal[Title/Abstract] OR Intestinal[Title/Abstract] OR Stool[Title/Abstract] OR Feacal[Title/Abstract] OR Fecal[Title/Abstract] OR Feces[Title/Abstract] |
| 10. 1 OR 2 |
| 11. 3 OR 4 |
| 12. 5 OR 6 |
| 13. 7 OR 8 |
| 14. 10 OR 11 OR 12 |
| 15. 9 AND 13 AND 14 |
| Results: 2628 |
|  |
| **Embase LIMIT: humans and English** |
| 1. 'attention deficit hyperactivity disorder'/exp |
| 2. 'adhd':ab,ti OR 'attention deficit':ab,ti OR 'disruptive behavior disorders':ab,ti OR 'attention deficit disorder':ab,ti OR 'attention deficit disorder with hyperactivity':ab,ti OR 'attention deficit hyperactivity disorder':ab,ti |
| 3. 'autism'/exp |
| 4. 'autism':ab,ti OR 'autism spectrum disorder':ab,ti OR 'autism, early infantile':ab,ti OR 'autism, infantile':ab,ti OR 'autistic child':ab,ti OR 'autistic children':ab,ti OR 'autistic disorder':ab,ti OR 'autistic spectrum disorder':ab,ti OR 'child development disorders, pervasive':ab,ti OR 'childhood autism':ab,ti OR 'classical autism':ab,ti OR 'early infantile autism':ab,ti OR 'infantile autism':ab,ti OR 'infantile autism, early':ab,ti OR 'kanner syndrome':ab,ti OR 'pervasive child development disorders':ab,ti OR 'pervasive developmental disorder':ab,ti OR 'pervasive developmental disorders':ab,ti OR 'typical autism':ab,ti |
| 5. 'tic '/exp |
| 6. 'facial twitching':ab,ti OR 'habit spasm':ab,ti OR 'nervous tic':ab,ti OR 'nervous twitch':ab,ti OR 'spasm, habit':ab,ti OR 'tic disorder':ab,ti OR 'tic disorders':ab,ti OR tics:ab,ti OR tic:ab,ti |
| 7. 'microbiome'/exp |
| 8. 'micro biome':ab,ti OR 'microbial biome':ab,ti OR 'microbiomes':ab,ti OR 'microbiome':ab,ti |
| 9. 'intestine'/exp |
| 10. 'bowel':ab,ti OR 'gut':ab,ti OR 'intestinal tract':ab,ti OR 'intestine lumen':ab,ti OR 'intestines':ab,ti OR 'intestinum':ab,ti OR 'intestine':ab,ti |
| 11. 1 OR 2 |
| 12. 3 OR 4 |
| 13. 5 OR 6 |
| 14. 7 OR 8 |
| 15. 9 OR 10 |
| 16. 11 OR 12 OR 13 |
| 17. 14 AND 15 AND 16 |
| Results: 750 |
|  |
| **Cochrane Library** |
| 1. “Attention deficit disorder with hyperactivity”[MeSH] |
| 2. Deficit-Hyperactivity Disorder or Attention or ADHD or ADDH or Disorder, Attention Deficit-Hyperactivity or Attention Deficit-Hyperactivity Disorder or Attention Deficit Hyperactivity Disorders or Deficit-Hyperactivity Disorders, Attention or Syndromes, Hyperkinetic or Attention Deficit Disorders with Hyperactivity or Attention Deficit Hyperactivity Disorder or Disorders, Attention Deficit-Hyperactivity or Attention Deficit-Hyperactivity Disorders or Hyperkinetic Syndrome or Attention Deficit Disorders or Deficit Disorders, Attention or Deficit Disorder, Attention or Disorders, Attention Deficit or Disorder, Attention Deficit or Attention Deficit Disorder or Minimal Brain Dysfunction or Dysfunction, Minimal Brain or Brain Dysfunction, Minimal |
| 3. “Autism Spectrum Disorder”[MeSH] |
| 4. Autistic Spectrum Disorder or Disorder, Autistic Spectrum or Autism Spectrum Disorders or Autistic Spectrum Disorders |
| 5. “Tourette syndrome” [MeSH] or “Tic Disorders”[MeSH] |
| 6. Chronic Motor and Vocal Tic Disorder or Tourettes Disease or Tourette's Disease or Gilles de la Tourette's Disease or Tourettes Syndrome or Tourettes Disorder or Multiple Motor and Vocal Tic Disorder, Combined or Tourette's Syndrome or Gilles de la Tourette Syndrome or Tic Disorder, Combined Vocal and Multiple Motor or Combined Multiple Motor and Vocal Tic Disorder or Gilles De La Tourette's Syndrome or Syndrome, Tourette or Combined Vocal and Multiple Motor Tic Disorder or Tourette's Disorder or Tourette Disease or Tourette Disorder or Gilles de la Tourette Disorder or Tic Disorder, Post Traumatic or Tic Disorder, Post-Traumatic or Post-Traumatic Tic Disorder or Post Traumatic Tic Disorder or Tic Disorders, Post-Traumatic or Post-Traumatic Tic Disorders or Tic Disorder, Motor or Motor Tic Disorder or Tic Disorders, Motor or Motor Tic Disorders or Tic Disorders, Vocal or Tic Disorder, Vocal or Vocal Tic Disorder or Vocal Tic Disorders or Tic Disorder or Chronic Motor or Vocal Chronic Motor or Vocal Tic Disorder or Motor, Vocal Tic Disorder or Chronic, Tic Disorder or Transient Tic Disorder or Tic Disorders, Transient or Transient Tic Disorders or Tic Disorder, Transient or Childhood Tic Disorders or Tic Disorder, Childhood or Childhood Tic Disorder or Tic Disorders, Childhood |
| 7. Microbiota[MeSH] |
| 8. Microbiomes or Microbiome or Microbiome, Human or Human Microbiomes or Human Microbiome or Microbial Communities or Composition, Microbial Community or Community Composition, Microbial or Microbial Community Compositions or Microbial Community Composition or Community, Microbial or Microbiotas or Microbial Community or Community Structure, Microbial or Microbial Community Structure or Microbial Community Structures |
| 9. Intestines[MeSH] |
| 10. Gut or Gastric or Gastrointestinal or Intestinal or Stool or Feacal or Fecal or Feces Bowel or Intestinum |
| 11. 1 or 2 |
| 12. 3 or 4 |
| 13. 5 or 6 |
| 14. 7 or 8 |
| 15. 9 or 10 |
| 16. 11 or 12 or 13 |
| 17. 14 and 15 and 16 |
| Results: 231 |
|  |
| **Web of Science LIMIT: English** |
| 1. Attention Deficit Disorder with Hyperactivity  (Topic) or ADHD (Topic) or ADDH (Topic) or Attention Deficit Disorders with Hyperactivity (Topic) or Attention Deficit Hyperactivity Disorder (Topic) or Attention Deficit-Hyperactivity Disorder (Topic) or Deficit-Hyperactivity Disorder, Attention (Topic) or Disorder, Attention Deficit-Hyperactivity (Topic) or Hyperkinetic Syndrome (Topic) or Syndromes, Hyperkinetic (Topic) or Attention Deficit Disorder (Topic) or Deficit Disorder, Attention (Topic) or Disorder, Attention Deficit (Topic) or Brain Dysfunction, Minimal (Topic) or Dysfunction, Minimal Brain (Topic) or Minimal Brain Dysfunction (Topic) |
| 2. Autism Spectrum Disorder (Topic) or Autism Spectrum Disorder (Topic) or Autism Spectrum Disorders (Topic) or Autistic Spectrum Disorder (Topic) or Autistic Spectrum Disorders (Topic) or Disorder, Autistic Spectrum (Topic) |
| 3. Tourette syndrome (Topic) or Tic Disorders (Topic)  or Tourette Disease (Topic) or Tourette Disorder (Topic) or Tic Disorder (Topic) or Tourette's disorder (Topic) or Syndrome, Tourette (Topic) |
| 4.Microbiota (Topic) or Microbiome (Topic) or Ecosystem (Topic) or Bacteria (Topic) or Flora (Topic) or Microflora (Topic) or Dysbiosis (Topic) |
| 5.Gut (Topic) or Gastrointestinal (Topic) or Intestinal (Topic) or Stool (Topic) or Feacal (Topic) or Fecal (Topic) or Feces (Topic) |
| 6. #1 OR #2 OR #3 |
| 7. #4 AND #5 AND #6 |
| Results: 1436 |
|  |
| **Scopus** **LIMIT: English** |
| 1.TITLE-ABS-KEY ( attention AND deficit AND disorder AND with AND hyperactivity ) OR TITLE-ABS-KEY ( adhd ) OR TITLE-ABS-KEY ( addh ) OR TITLE-ABS-KEY ( attention AND deficit AND disorders AND with AND hyperactivity ) OR TITLE-ABS-KEY ( attention AND deficit AND hyperactivity AND disorder ) OR TITLE-ABS-KEY ( attention AND deficit-hyperactivity AND disorder ) OR TITLE-ABS-KEY ( deficit-hyperactivity AND disorder, AND attention ) OR TITLE-ABS-KEY ( disorder, AND attention AND deficit-hyperactivity ) OR TITLE-ABS-KEY ( hyperkinetic AND syndrome ) OR TITLE-ABS-KEY ( syndromes, AND hyperkinetic ) OR TITLE-ABS-KEY ( attention AND deficit AND disorder ) OR TITLE-ABS-KEY ( deficit AND disorder AND attention ) OR TITLE-ABS-KEY ( disorder AND attention AND deficit ) OR TITLE-ABS-KEY ( brain AND dysfunction, AND minimal ) OR TITLE-ABS-KEY ( dysfunction AND minimal AND brain ) OR TITLE-ABS-KEY ( minimal AND brain AND dysfunction ) |
| 2.TITLE-ABS-KEY ( autism AND spectrum AND disorder ) OR TITLE-ABS-KEY ( autism AND spectrum AND disorders ) OR TITLE-ABS-KEY ( autistic AND spectrum AND disorder ) OR TITLE-ABS-KEY ( autistic AND spectrum AND disorders ) OR TITLE-ABS-KEY ( disorder AND autistic AND spectrum ) |
| 3.TITLE-ABS-KEY ( tourette AND syndrome ) OR TITLE-ABS-KEY ( tic AND disorders ) OR TITLE-ABS-KEY ( tourette AND disease ) OR TITLE-ABS-KEY ( tourette AND disorder* ) OR TITLE-ABS-KEY ( tic AND disorder* ) OR TITLE-ABS-KEY ( tourette's AND disorder ) OR TITLE-ABS-KEY ( syndrome AND tourette ) |
| 4.TITLE-ABS-KEY ( microbiota ) OR TITLE-ABS-KEY ( microbiome ) OR TITLE-ABS-KEY ( ecosystem ) OR TITLE-ABS-KEY ( bacteria ) OR TITLE-ABS-KEY ( flora ) OR TITLE-ABS-KEY ( microflora ) OR TITLE-ABS-KEY ( dysbiosis ) |
| 5.TITLE-ABS-KEY ( gut ) OR TITLE-ABS-KEY ( gastrointestinal ) OR TITLE-ABS-KEY ( intestinal ) OR TITLE-ABS-KEY ( stool ) OR TITLE-ABS-KEY ( feacal ) OR TITLE-ABS-KEY ( fecal ) OR TITLE-ABS-KEY ( feces ) |
| 6. 1 OR 2 OR 3 |
| 7. 4 AND 5 AND 6 |
| Results: 1516 |
|  |
| **PsycINFO LIMIT: English** |
| 1. TX Attention Deficit Disorder OR TX Attention Deficit Disorder with Hyperactivity OR TX Attention Span OR TX Distractibility OR TX Hyperactivity OR TX Impulsiveness OR TX Mental Disorders OR TX Neurodiversity OR TX Oppositional Defiant Disorder |
| 2. TX Autism Spectrum Disorders OR TX Antisocial Personality Disorder OR TX Applied Behavior Analysis OR TX Rett Syndrome OR TX Stereotyped Behavior OR TX Stereotypic Movement Disorder |
| 3. TX Tourette syndrome OR TX Tic Disorders OR TX Echolalia OR Tics |
| 4. TX Gastrointestinal Microbiota OR TX Gastrointestinal System OR TX Microorganisms OR TX Gut-Brain Axis |
| 5. 1 OR 2 OR 3 |
| 6. 4 AND 5 |
| Results: 498 |
