# Supplementary material for: Alterations in gut microbiota composition in neurodevelopmental disorders: a systematic review and meta-analysis
Source: Front Microbiol. 2025 Dec 9;16:1650212. doi: 10.3389/fmicb.2025.1650212 (PMC12723412; doi:10.3389/fmicb.2025.1650212)
Supplement: Supplementary file 8 [file Table_3.DOCX]

**Table S3.** Stool sample collection, storage and DNA extraction procedures of the included studies.

| **Study** | **Disorder** | **Collection & handling by participant** | **Long-term storage** | **DNA extraction method** |
| --- | --- | --- | --- | --- |
| Wang et al.2011 | ASD | Collected from participants over a 48-h period, then frozen immediately in a portable freezer and stored at 20°C | At −80 °C until use | Repeat bead beating plus column method |
| Kang et al.2013 | ASD | Frozen fecal samples were shipped overnight to Arizona State University with a cold pack | At −80 °C until use | QIAamp (Qiagen, CA) DNA Stool Mini Kit |
| Strati et al.2017 | ASD | Collected and aliquoted based on the Rome III constipation criteria | At −80 °C until use | FastDNA™ SPIN Kit (MP Biomedicals, Santa Ana, CA, USA) |
| Pulikkan et al.2018 | ASD | Collected after breakfast | At −80°C within 2 hours | QIAamp Stool Mini Kit (Qiagen, CA, USA) |
| Zhang et al.2018 | ASD | Collected in the homes of the participants by their parents. | At −80 °C until use | Qubit dsDNA HS Assay Kit |
| Coretti et al.2018 | ASD | Collected in sterile vials | At −80 °C until use | QIAamp DNA Stool Mini Kit (Qiagen) |
| Sun et al.2019 | ASD | Placed in a sterile stool container, frozen immediately in liquid nitrogen | At −80 °C until use | QIAamp DNA Stool  Mini Kit (Qiagen, Valencia, California) |
| Plaza-Díaz et al.2019 | ASD | Collected in plastic sterile containers | At −80 °C until use | QIAamp DNA Stool Mini Kit (QIAGEN, Barcelona, Spain) |
| Ma et al.2019 | ASD | Collected stool samples in a sterile container, refrigerate, and transport within 12 hours with ice packs. | At -80°C within 10 min, until use | QIAamp Fast DNA Stool  Mini kit (Qiagen, Hilden, Germany) |
| Niu et al.2019 | ASD | Collected at home by their parents | - | OMEGA E.Z.N.A Stool DNA Kit and were quantified by Nanodrop. |
| Zou et al.2020 | ASD | Collected in the homes and transferred to the laboratory within three hours | At −80 °C until use | QIAamp DNA Stool Mini Kit |
| Ding et al.2020 | ASD | Collected during the day and transported to the  laboratory for processing within 30 min. | At −80 °C until use | Cetyltrimethylammonium bromide (CTAB)-based method |
| Kovtun et al.2020 | ASD | - | At −80 °C until use | QIAamp Fast DNA Stool Mini Kit (Qiagen, Germany) |
| Chen et al.2020 | ASD | Collected at home using DNA-stabilizing tubes, which keep gut bacteria intact at room temperature for 24 hours | At −80 °C until use | MiSeq reagent kit v3 (600-cycle) (MS-102-3033; Illumina, USA) |
| Cao et al.2021 | ASD | Collected with the MGIEasy Fecal DNA Kit (MGI Tech) and stored at room temperature until processing | At −80 °C until use | DNeasy PowerSoil Kit (QIAGEN) |
| Wan et al. 2022 | ASD | Collected in MGIEasy DNA kits and stored at room temperature | At −80 °C until use | Maxwell RSC PureFood GMO and Authentication Kit (Promega) |
| Ye et al.2021 | ASD | Collected with sterilized 2-ml tubes containing pure ethanol, aliquoted | At −80 °C until use | PSP Spin StooL DNA Kit/PSP Spin StooL  DNA PLus Kit |
| Huang et al.2021 | ASD | Collected stool samples using a kit and kept at room temperature for 2 days | At −80 °C until use | Stool DNA extraction kit (TIANGEN Biotech, DP712) |
| Chen et al.2021 | ASD | Collected by their parents and kept in 1.8 ml sterile microcentrifuge tubes | At −80 °C until use | QIAamp DNA Stool Mini Kit (Qiagen, Hilden, Germany) |
| Ding et al.2021 | ASD | Collected 5-10 g of feces into a sterile 50mL tube, stored in the patient's fridge at -20°C | At −80 °C until use | OMEGA-soil DNA Kit |
| Chen et al.2022 | ASD | Frozen at -20°C for up to seven days and transported in a dry ice box to the lab within two hours | At −80 °C until use | Column-based method (e.g. QIAamp PowerFecal DNA Kit, Qiagen) |
| Deng et al.2022 | ASD | Collected at home in the morning or on the night before and stored at −18° | At −80°C within 6 hours | The cetyl trimethyl ammonium bromide/sodium dodecyl sulfate method |
| Chiappori et al.2022 | ASD | Collected in stool nucleic acid collection and  transport tubes | At −80 °C until use | commercial kit and the relative protocol for pathogen detection (QiAamp DNA stool mini kit, Qiagen GmbH, Hilden, Germany) |
| He et al.2023 | ASD | Collected in sterile plastic cups | At −80°C within 1 hour | QIAamp DNA Stool Mini Kit (Qiagen, Hilden, Germany) |
| Bundgaard-Nielsen et al. 2023 | ASD | Collected and frozen at -20°C for up to 3 days, then shipped cold to the hospital labs | At −80 °C until use | QIAamp Powerfecal DNA kit (QIAGEN®, Cat. No. 51804) |
| Wang et al.2023 | ASD | Collected within 30 min after defecation | 200 mg of each was mixed with a special solution to keep the microbes stable at room temperature for 6 month | QIAamp DNA Stool Mini Kit (Qiagen, Hilden, Germany) |
| Zhao et al.2023 | ASD | Collected as instructed, and transported at low temperatures immediately after collection | At −80 °C until use | DNA Kit (Omega Bio-tek, GA, U.S.) |
| Mendive Dubourdieu et al.2023 | ASD | Collected a stool sample, then refrigerated and delivered it to the clinic within 48 hours | At −80 °C until use | Quick–DNA Fecal/Soil Microbe Miniprep Kit (Zymo Research—Catalog  No. D6010). |
| Pang et al.2023 | ASD | Collected in MicroLockerT preservation solution and transferred to the lab within three hours at room temperature. | - | QIAamp DNA Stool Mini Kit (QIAGEN, Hilden, Germany) |
| Yitik Tonkaz et al.2023 | ASD | Stored in a freezer and brought to the hospital inside cool-packs within 12 h | At −80 °C until use | - |
| Xu et al.2023 | ASD | Collected and temporarily stored in a refrigerator at -20°C | At -80°C within 7 days, until use | - |
| Li et al.2024 | ASD | Collected according to the instructions and delivered immediately at a low temperature | At −80 °C until use | DNA extraction kit (DP712, Tiangen Company, Beijing, China) |
| Bhusri et al.2025 | ASD | Shipped to the lab within 1 month and kept stable at room temperature using DNA/RNA Shield | At −20 °C until use | QIAamp Fast DNA Stool Mini Kit (Qiagen, Hilden, Germany) |
| Aarts et al.2017 | ADHD | Stored at 4˚C straight after collection | At -80˚C within 24 hours, until use | DNeasy1 Blood and Tissue Kit (Qiagen, Venlo, The Netherlands) |
| Jiang et al.2018 | ADHD | Collected in a sterile cup and froze at -20°C at home, kept in an icebox and delivered to the laboratory within 30 min | At −80 °C until use | QIAamp DNA Stool Mini Kit (Qiagen; Hilden, Germany) |
| Prehn-Kristensen et al. 2018 | ADHD | Collected in Sarstedt fecal collection tubes (Nu¨mbrecht, Germany) and stored at 4˚C until preparation | At −80 °C until use | FastDNA^TM^ SPIN KIT FOR SOIL (Qbiogene, Carlsbad, CA, USA) |
| Szopinska-Tokov et al.2020 | ADHD | Collected at home and stored at 4 ˚C | At -80°C within 24 hours, until use | Repeated bead-beating step and the Maxwellfi16 Instrument (Promega, Leiden, The Netherlands) |
| Wang et al.2020 | ADHD | Frozen at -20°C for up to seven days, then transported in a dry ice box to the lab within two hours | At −80 °C until use | QIAamp® DNA Stool Mini Kit (QIAGEN, Tokyo, Japan) |
| Wan et al.2020 | ADHD | Collected at 8:00 am in the Pediatric Outpatient Department and stored in a sterile plastic cup | At −80 °C until use | HiPure Stool DNA kits (Angen Biotech Co., Ltd., Guangzhou, China) |
| Richarte et al.2021 | ADHD | Collected at home, stabilized with the  OMNIgene.GUT (OM-200) (DNA Genotek Inc.) kit, and then transported to the laboratory | At −80 °C until use | QIAamp® PowerFecal® DNA extraction kit (QIAgen, Hilden, Germany) |
| Steckler et al. 2024 | ADHD | Collected from all studied subjects, refrigerated, and kept in ice packs | At −80 °C until use | Dneasy PowerSoil Kit (Qiagen, Hilden, Germany) |
| Panpetch et al.2024 | ADHD | Collected in sealed sterile tubes, which were immediately frozen at home | At −80 °C until use | QIAamp®Fast DNA stool mini kit (Qiagen, Hilden, Germany) |
| Boonchooduang et al.2025 | ADHD | Collected fecal samples in sterile plastic cups and refrigerated them at 4 °C immediately | At −80 °C until use | QIAamp PowerFecal Pro DNA kit (Qiagen, Germany) |
| Wang et al.2022 | TD | Collected into three 2 mL sterile tubes | At -80°C within 30 min, until use | The E.Z.N.A. R@ Soil DNA Kit (Omega Biotek) |
| Bao et al.2023 | TD | Collected within 3 minutes, sealed, and shipped cold to the lab within 2 days | At −80 °C until use | QIAamp PowerFecal Pro DNA Kit (QIAGEN, Germany) |
