# Supplementary material for: Alterations in gut microbiota composition in neurodevelopmental disorders: a systematic review and meta-analysis
Source: Front Microbiol. 2025 Dec 9;16:1650212. doi: 10.3389/fmicb.2025.1650212 (PMC12723412; doi:10.3389/fmicb.2025.1650212)
Supplement: Supplementary file 9 [file Table_4.DOCX]

**Table S4.** Results of the meta-analysis at phylum, family, and genus levels in NDD children

and controls, and the significance of the difference between them.

|  | Studies  Included | I^2^ (%) | SMD (95%CI) | Z | | *P* |
| --- | --- | --- | --- | --- | --- | --- |
| **Actinobacteria** | 10 | 81.6 | -0.09(-0.45,0.28) | | -0.458 | 0.647 |
| **Bacteroidetes** | 11 | 74.7 | 0.04(-0.26,0.35) | | 0.270 | 0.787 |
| **Firmicutes** | 10 | 76.1 | -0.05(-0.36,0.26) | | -0.323 | 0.747 |
| **Proteobacteria** | 8 | 87.6 | -0.11(-0.60,0.37) | | -0.452 | 0.651 |
| **Verrucomicrobia** | 4 | 0 | -0.10(-0.31,0.11) | | -0.915 | 0.360 |
| Acidaminococcaceae | 4 | 87.3 | 0.01(-0.61,0.64) | | 0.045 | 0.964 |
| Bacteroidaceae | 5 | 89.7 | -0.12(-0.80,0.56) | | -0.351 | 0.726 |
| Bifidobacteriaceae | 5 | 63.6 | 0.24(-0.08,0.55) | | 1.470 | 0.142 |
| Enterobacteriaceae | 4 | 86.7 | -0.18(-0.94,0.58) | | -0.472 | 0.637 |
| Lachnospiraceae | 4 | 59.9 | -0.04(-0.34,0.26) | | -0.272 | 0.785 |
| Pasteurellaceae | 4 | 85.6 | 0.36(-0.25, 0.98) | | 1.161 | 0.246 |
| Peptostreptococcaceae | 4 | 68.7 | 0.47(0.05,0.90) | | 2.192 | 0.028 |
| Prevotellaceae | 6 | 73.4 | 0.18(-0.20,0.57) | | 0.939 | 0.348 |
| Rikenellaceae | 5 | 20.4 | -0.01(-0.21,0.19) | | -0.067 | 0.947 |
| Ruminococcaceae | 6 | 62.8 | -0.20(-0.50,0.11) | | -1.253 | 0.210 |
| Veillonellaceae | 6 | 76.3 | 0.10(-0.30,0.50) | | 0.492 | 0.623 |
| *Akkermansia* | 8 | 60.1 | 0.05(-0.22,0.32) | | 0.367 | 0.713 |
| *Alistipes* | 7 | 75.1 | -0.19(-0.51,0.14) | | -1.142 | 0.253 |
| *Bacteroides* | 16 | 87.7 | 0.03(-0.30,0.36) | | 0.180 | 0.858 |
| *Bifidobacterium* | 12 | 83 | -0.16(-0.49,0.17) | | -0.968 | 0.333 |
| *Blautia* | 9 | 87.9 | -0.17(-0.56,0.22) | | -0.836 | 0.403 |
| *Clostridium* | 3 | 0 | 0.17(-0.09,0.44) | | 1.284 | 0.199 |
| *Collinsella* | 5 | 74.2 | 0.16(-0.20,0.52) | | 0.888 | 0.374 |
| *Coprococcus* | 9 | 75.3 | -0.27(-0.61,0.06) | | -1.623 | 0.105 |
| *Desulfovibrio* | 6 | 64.7 | 0.07(-0.27,0.41) | | 0.420 | 0.674 |
| *Dialister* | 6 | 74.2 | 0(-0.38,0.37) | | -0.021 | 0.983 |
| *Dorea* | 4 | 85.2 | 0.10(-0.56,0.76) | | 0.301 | 0.673 |
| *Enterococcus* | 4 | 18.3 | 0.04(-0.20,0.28) | | 0.349 | 0.727 |
| *Escherichia/Shigella* | 6 | 33.7 | -0.39(-0.59,-0.19) | | -3.799 | <0.001 |
| *Eubacterium* | 5 | 34.8 | 0.33(0.20,0.47) | | 4.776 | <0.001 |
| *Faecalibacterium* | 12 | 78.9 | 0.12(-0.15,0.39) | | 0.902 | 0.367 |
| *Fusobacterium* | 3 | 67.7 | 0.17(-0.36,0.70) | | 0.626 | 0.531 |
| *Lachnoclostridium* | 5 | 88.3 | 0.14(-0.36,0.65) | | 0.557 | 0.578 |
| *Lactobacillus* | 8 | 69.6 | 0.07(-0.25,0.39) | | 0.423 | 0.672 |
| *Megamonas* | 6 | 77.7 | 0.13(-0.21,0.46) | | 0.725 | 0.468 |
| *Parabacteroides* | 9 | 68.1 | -0.27(-0.57,0.04) | | -1.718 | 0.086 |
| *Phascolarctobacterium* | 3 | 88.1 | 0.10(-0.78,0.98) | | 0.226 | 0.821 |
| *Prevotella* | 10 | 73.9 | -0.03(-0.31,0.25) | | -0.197 | 0.844 |
| *Roseburia* | 6 | 69 | -0.39(-0.78,0) | | -1.968 | 0.049 |
| *Ruminococcus* | 9 | 70.2 | -0.07(-0.36,0.22) | | -0.467 | 0.641 |
| *Streptococcus* | 5 | 60 | 0.13(--0.20,0.46) | | 0.749 | 0.454 |
| *Sutterella* | 8 | 79.4 | -0.31(-0.69,0.08) | | -1.575 | 0.115 |
| *Veillonella* | 4 | 77.2 | -0.24(-0.79,0.30) | | -0.882 | 0.378 |
