# Supplementary material for: Alterations in gut microbiota composition in neurodevelopmental disorders: a systematic review and meta-analysis
Source: Front Microbiol. 2025 Dec 9;16:1650212. doi: 10.3389/fmicb.2025.1650212 (PMC12723412; doi:10.3389/fmicb.2025.1650212)
Supplement: Supplementary file 10 [file Table_5.DOCX]

**Table S5.** NOS score for Assessment of Quality of Included Studies.

| Study (year) | Disorder | | Selection | | | | | Comparability | | | Exposure | | | Overall  score |
| --- | --- | --- | --- | --- | --- | --- | --- | --- | --- | --- | --- | --- | --- | --- |
|  |  | | Definition  adequate | Representativeness  of the cases | | Selection  of controls | Definition  of controls | | Comparability  of cases and  controls | Ascertainment  of exposure | | Same method  of ascertainment  for cases and  controls | Nonresponse  rate |  |
| Wang et al.2011  Kang et al.2013  Strati et al.2017  Pulikkan et al.2018  Zhang et al.2018  Coretti et al.2018  Sun et al.2019  Plaza-Díaz et al.2019  Ma et al.2019  Niu et al.2019  Zou et al.2020  Ding et al.2020  Kovtun et al.2020  Chen et al.2020  Cao et al.2021  Wan et al. 2022  Ye et al.2021  Huang et al.2021  Chen et al.2021  Ding et al.2021  Chen et al.2022  Deng et al.2022  Chiappori et al.2022  He et al.2023  Bundgaard-Nielsen et al. 2023  Wang et al.2023  Zhao et al.2023  Mendive Dubourdieu et al.2023  Pang et al.2023  Yitik Tonkaz et al.2023  Xu et al.2023  Li et al.2024  Bhusri et al.2025  Aarts et al.2017  Jiang et al.2018  Prehn-Kristensen et al. 2018  Szopinska-Tokov et al.2020  Wang et al.2020  Wan et al.2020  Richarte et al.2021  Steckler et al. 2024  Panpetch et al.2024  Boonchooduang et al.2025  Wang et al.2022  Bao et al.2023 | | ASD  ASD  ASD  ASD  ASD  ASD  ASD  ASD  ASD  ASD  ASD  ASD  ASD  ASD  ASD  ASD  ASD  ASD  ASD  ASD  ASD  ASD  ASD  ASD  ASD  ASD  ASD  ASD  ASD  ASD  ASD  ASD  ASD  ADHD  ADHD  ADHD  ADHD  ADHD  ADHD  ADHD  ADHD  ADHD  ADHD  TD  TD | 1  1  1  1  1  1  1  1  1  1  1  1  1  1  1  1  1  1  1  1  1  1  1  1  1  1  1  1  1  1  1  1  1  1  1  1  1  1  1  1  1  1  1  1  1 | 1  1  1  1  1  1  0  1  1  1  1  1  1  1  1  1  1  1  1  1  1  1  1  1  1  1  1  1  0  0  1  1  1  1  1  1  1  1  1  0  1  1  1  0  1 | 1  1  1  1  1  1  1  1  1  1  1  1  1  1  1  1  1  1  1  1  1  1  1  1  1  1  1  1  1  1  1  1  0  1  1  1  1  1  1  1  1  1  1  1  1 | | 1  1  1  1  1  1  1  1  1  1  1  1  1  1  1  1  1  1  1  1  1  1  1  1  0  1  1  1  1  0  1  1  1  1  1  1  1  1  1  1  1  1  1  1  1 | | 1  1  1  1  1  1  1  2  2  1  1  2  1  2  2  2  2  2  1  1  2  1  1  2  1  1  1  2  1  1  1  1  1  1  1  1  1  1  1  1  1  2  1  1  1 | 1  1  1  1  1  1  1  1  1  1  1  1  1  1  1  1  1  1  1  1  1  1  1  1  1  1  1  1  1  1  1  1  1  1  1  1  1  1  1  1  1  1  1  1  1 | | 1  1  1  1  1  1  1  1  1  1  1  1  1  1  1  1  1  1  1  1  1  1  1  1  1  1  1  1  1  1  1  1  1  1  1  1  1  1  1  1  1  1  1  1  1 | 1  1  1  1  1  1  1  1  1  1  1  1  1  1  1  1  1  1  1  1  1  1  1  1  1  1  1  1  1  1  1  1  1  1  1  1  1  1  1  1  1  1  1  1  1 | 8  8  8  8  8  8  7  9  9  8  8  9  8  9  9  9  9  9  8  8  9  8  8  9  7  8  8  9  7  6  8  8  7  8  8  8  8  8  8  7  8  9  8  7  8 |

The quality of the studies was evaluated using the Newcastle–Ottawa Quality Assessment Scale for observational studies. ASD, autism spectrum disorder; ADHD, attention deficit hyperactivity disorder; TD, tic disorder. High Quality: ≥7 scores; Moderate Quality: 5-6 scores; Low Quality: ≤4 scores.
