# Supplementary material for: Alterations in gut microbiota composition in neurodevelopmental disorders: a systematic review and meta-analysis
Source: Front Microbiol. 2025 Dec 9;16:1650212. doi: 10.3389/fmicb.2025.1650212 (PMC12723412; doi:10.3389/fmicb.2025.1650212)
Supplement: Supplementary file 11 [file Table_6.DOCX]

## Table S6.1 Egger’s tests for publication bias of Alpha diversity indexes.

| Alpha | *P* for Egger’s test |
| --- | --- |
| Chao1 | 0.099 |
| Shannon | 0.184 |
| Simpson | 0.710 |
| ACE | 0.683 |
| Observed species | 0.289 |

No significant publication bias is present when *P* > 0.05.

## Table S6.2 Egger’s tests for publication bias of gut microbiota at the phylum level.

| Phylum | *P* for Egger’s test |
| --- | --- |
| **Actinobacteria** | 0.591 |
| **Bacteroidetes** | 0.218 |
| **Firmicutes** | 0.584 |
| **Proteobacteria** | 0.491 |
| **Verrucomicrobia** | 0.255 |

No significant publication bias is present when *P* > 0.05.

## Table S6.3 Egger’s tests for publication bias of gut microbiota at the family level.

| Genus | *P* for Egger’s test |
| --- | --- |
| Acidaminococcaceae | 0.398 |
| Bacteroidaceae | 0.622 |
| Bifidobacteriaceae | 0.518 |
| Enterobacteriaceae | 0.319 |
| Lachnospiraceae | 0.862 |
| Pasteurellaceae | 0.277 |
| Peptostreptococcaceae | 0.174 |
| Prevotellaceae | 0.517 |
| Rikenellaceae | 0.627 |
| Ruminococcaceae | 0.919 |
| Veillonellaceae | 0.795 |

No significant publication bias is present when *P* > 0.05.

## sTable6.4 Egger’s tests for publication bias of gut microbiota at the genus level.

| Genus | *P* for Egger’s test |
| --- | --- |
| *Akkermansia* | 0.189 |
| *Alistipes* | 0.112 |
| *Bacteroides* | 0.225 |
| *Bifidobacterium* | 0.968 |
| *Blautia* | 0.187 |
| *Clostridium* | 0.942 |
| *Collinsella* | 0.830 |
| *Coprococcus* | 0.766 |
| *Desulfovibrio* | 0.468 |
| *Dialister* | 0.403 |
| *Dorea* | 0.035 |
| *Enterococcus* | 0.578 |
| *Escherichia/Shigella* | 0.507 |
| *Eubacterium* | 0.544 |
| *Faecalibacterium* | 0.947 |
| *Fusobacterium* | 0.619 |
| *Lachnoclostridium* | 0.875 |
| *Lactobacillus* | 0.584 |
| *Megamonas* | 0.375 |
| *Parabacteroides* | 0.084 |
| *Phascolarctobacterium* | 0.056 |
| *Prevotella* | 0.633 |
| *Roseburia* | 0.577 |
| *Ruminococcus* | 0.768 |
| *Streptococcus* | 0.881 |
| *Sutterella* | 0.667 |
| *Veillonella* | 0.166 |

No significant publication bias is present when *P* > 0.05.
