# Supplementary material for: Alterations in gut microbiota composition in neurodevelopmental disorders: a systematic review and meta-analysis
Source: Front Microbiol. 2025 Dec 9;16:1650212. doi: 10.3389/fmicb.2025.1650212 (PMC12723412; doi:10.3389/fmicb.2025.1650212)
Supplement: Supplementary file 12 [file Table_7.DOCX]

**Table S7.** Methodology and findings of the included studies assessing

beta diversity for the patient vs. control group comparison.

| **Study** | **Disorder** | **Metric** | **Analysis** | **Finding** |
| --- | --- | --- | --- | --- |
| Wang et al.2011 | ASD | - | - | - |
| Kang et al.2013 | ASD | Weighted Unifrac | PCoA | sig. different |
| Strati et al.2017 | ASD | Unweighted Unifrac,  Weighted Unifrac,  Bray-Curtis | PCoA  PERMANOVA | sig. different  sig. different  sig. different |
| Pulikkan et al.2018 | ASD | Unweighted Unifrac | PCoA  PERMANOVA | sig. different  sig. different |
| Zhang et al.2018 | ASD | Weighted Unifrac, Bray-Curtis | PCoA | no sig. difference  sig. different |
| Coretti et al.2018 | ASD | Unweighted Unifrac  Weighted Unifrac | PCoA | sig. different  sig. different |
| Sun et al.2019 | ASD | - | - | - |
| Plaza-Díaz et al.2019 | ASD | - | - | - |
| Ma et al.2019 | ASD | Unweighted Unifrac,  Weighted Unifrac,  Bray-Curtis | PCoA | sig. different  no sig. difference  sig. different |
| Niu et al.2019 | ASD | - | - | - |
| Zou et al.2020 | ASD | Weighted Unifrac | - | sig. different |
| Ding et al.2020 | ASD | Unweighted Unifrac,  Weighted Unifrac,  Bray-Curtis | - | sig. different  no sig. difference  sig. different |
| Kovtun et al.2020 | ASD | - | - | - |
| Chen et al.2020 | ASD | Bray-Curtis | PCoA  PERMANOVA | sig. different |
| Cao et al.2021 | ASD | Bray-Curtis | - | - |
| Wan et al. 2022 | ASD | Bray-Curtis | PCoA  PERMANOVA | sig. different |
| Ye et al.2021 | ASD | Weighted Unifrac | PCoA | sig. different |
| Huang et al.2021 | ASD | Unweighted Unifrac,  Weighted Unifrac,  Bray-Curtis | PERMANOVA | sig. different  sig. different  sig. different |
| Chen et al.2021 | ASD | Unweighted Unifrac,  Weighted Unifrac,  Bray-Curtis | PCoA | no sig. difference  no sig. difference  no sig. difference |
| Ding et al.2021 | ASD | Unweighted Unifrac | PCoA | no sig. difference |
| Chen et al.2022 | ASD | Unweighted Unifrac  Weighted Unifrac | - | no sig. difference  sig. different |
| Deng et al.2022 | ASD | Bray-Curtis | ANOSIM | sig. different |
| Chiappori et al.2022 | ASD | - | - | - |
| He et al.2023 | ASD | - | PCoA  PERMANOVA | sig. different |
| Bundgaard-Nielsen et al. 2023 | ASD | Unweighted Unifrac,  Weighted Unifrac,  Bray-Curtis | PCoA  PERMANOVA | sig. different  sig. different  sig. different |
| Wang et al.2023 | ASD | - | PCoA  PERMANOVA | sig. different |
| Zhao et al.2023 | ASD | Unweighted Unifrac,  Weighted Unifrac,  Bray-Curtis | PCoA  PERMANOVA | sig. different  sig. different  sig. different |
| Mendive Dubourdieu et al.2023 | ASD | - | - | - |
| Pang et al.2023 | ASD | Bray-Curtis | PCoA | sig. different |
| Yitik Tonkaz et al.2023 | ASD | - | - | - |
| Xu et al.2023 | ASD | - | - | - |
| Li et al.2024 | ASD | Unweighted Unifrac,  Weighted Unifrac,  Bray-Curtis | PCoA  PERMANOVA | sig. different  sig. different  sig. different |
| Bhusri et al.2025 | ASD | Bray-Curtis | PCoA  PERMANOVA | no sig. difference |
| Aarts et al.2017 | ADHD | - | - | - |
| Jiang et al.2018 | ADHD | Unweighted Unifrac,  Weighted Unifrac,  Bray-Curtis | PCoA | no sig. difference  no sig. difference  no sig. difference |
| Prehn-Kristensen et al. 2018 | ADHD | - | ANOSIM,  ADONIS | sig. different |
| Szopinska-Tokov et al.2020 | ADHD | Weighted Unifrac | PCoA | sig. different |
| Wang et al.2020 | ADHD | Unweighted Unifrac,  Weighted Unifrac, | PCoA | no sig. difference  no sig. difference |
| Wan et al.2020 | ADHD | - | PCoA PERMANOVA | - |
| Richarte et al.2021 | ADHD | Unweighted Unifrac,  Weighted Unifrac,  Bray-Curtis | PCoA PERMANOVA | no sig. difference  no sig. difference  no sig. difference |
| Steckler et al. 2024 | ADHD | Unweighted Unifrac,  Weighted Unifrac | - | no sig. difference  no sig. difference |
| Panpetch et al.2024 | ADHD | Unweighted Unifrac,  Weighted Unifrac,  Bray-Curtis | PERMANOVA | no sig. difference  no sig. difference  no sig. difference |
| Boonchooduang et al.2025 | ADHD | Unweighted Unifrac,  Weighted Unifrac,  Bray-Curtis,  Jaccard | PCoA PERMANOVA | sig. different  sig. different  no sig. difference  no sig. difference |
| Wang et al.2022 | TD | - | PCoA  ANOSIM | sig. different |
| Bao et al.2023 | TD | Weighted Unifrac  Bray-Curtis | PCoA PERMANOVA | sig. different  sig. different |

ASD, autism spectrum disorder; ADHD, attention deficit hyperactivity disorder; TD, tic disorder;

PCoA, principal coordinates analysis; PERMANOVA, permutational analysis of variance; ANOSIM, analysis of similarities; ADONIS, permutational multivariate analysis of variance using distance matrices.
